# The histological growth patterns in liver metastases from colorectal cancer display differences in lymphoid, myeloid, and mesenchymal cells

**DOI:** 10.1002/mco2.70000

**Published:** 2024-11-19

**Authors:** Gemma Garcia‐Vicién, Núria Ruiz, Patrick Micke, José Carlos Ruffinelli, Kristel Mils, María Bañuls, Natalia Molina, Miguel A. Pardo, Laura Lladó, Artur Mezheyeuski, David G. Molleví

**Affiliations:** ^1^ Tumoural and Stromal Chemoresistance Group, Oncobell Program, IDIBELL L'Hospitalet de Llobregat Barcelona Catalonia Spain; ^2^ Department of Pathology Hospital Universitari de Bellvitge L'Hospitalet de Llobregat Barcelona Catalonia Spain; ^3^ Department of Immunology, Genetics and Pathology Uppsala University Uppsala Sweden; ^4^ Department of Medical Oncology Institut Català d'Oncologia, L'Hospitalet de Llobregat Barcelona Catalonia Spain; ^5^ Department of Surgery Hospital Universitari de Bellvitge L'Hospitalet de Llobregat Barcelona Catalonia Spain; ^6^ Program Against Cancer Therapeutic Resistance (ProCURE) Institut Català d'Oncologia, L'Hospitalet de Llobregat Barcelona Catalonia Spain; ^7^ Molecular Oncology Group, Vall d'Hebron Institute of Oncology Barcelona Catalonia Spain

**Keywords:** capsule, desmoplasia, hepatic metastases, histologic growth pattern, microenvironment

## Abstract

Colorectal liver metastases grow following different histologic growth patterns (HGPs), classified as desmoplastic and nondesmoplastic (dHGP, non‐dHGP), being the latter associated with worst prognosis. This study aimed to investigate the tumor microenvironment (TME) between HGPs supporting different survival. Multiplexed immunohistochemical staining was performed with the Opal7 system in a 100‐patients cohort to evaluate the tumor–liver interface with three different cell panels: lymphoid, myeloid, and carcinoma‐associated fibroblasts. Differences between HGPs were assessed by Mann–Whitney *U* test with Pratt correction and Holm–Bonferroni multitest adjustment. Cytotoxic T‐cells were more abundant in tumoral areas of dHGP, while non‐dHGP had higher macrophages infiltration, Th2, CD163^+^, and Calprotectin^+^ cells as well as higher pSMAD2 expression. Regarding carcinoma‐associated fibroblasts, several subsets expressing COL1A1 were enriched in dHGP, while αSMA^low^_single cells were present at higher densities in non‐dHGP. Interestingly, Calprotectin^+^ cells confer better prognoses in non‐dHGP, identifying a subgroup of good outcome patients that unexpectedly also show an enrichment in other myeloid cells. In summary, our results illustrate different TME landscapes with respect to HGPs. dHGP presents a higher degree of immunocompetence, higher amounts of Collagen 1 as well as lesser presence of myeloid cell populations, features that might be influencing on the better prognosis of encapsulated metastases.

## Graphical abstract

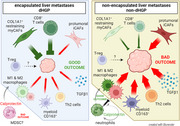

## 1 INTRODUCTION

Colorectal cancer (CRC) is the third most frequent tumor worldwide, with almost 2 million new cases annually (Global Cancer Observatory, https://gco.iarc.fr/). The annual death rate is roughly half the incidence, making it the leading global cause of death by cancer. Unfortunately, 50% of CRC patients develop liver metastases (CRCLM),^1^ with approximately 65–70% of them not candidates for surgery, with a life expectancy of <10% at 5 years, roughly.^2^ Likewise, treatment options for these nonoperable patients are limited, based mainly on chemotherapy combinations,^3^ with or without targeted therapies^4^, ^5^ or immunotherapy for microsatellite instability tumors.^6^ The prognosis is certainly much more encouraging in the 20% of patients who are initially candidates for surgery, reaching 35% with neoadjuvant therapy, with 5‐year survival rate of 60–65%.^4^

In the last decade, immunotherapy has revolutionized cancer therapy, shedding light on the different facets of the tumor microenvironment (TME), which is crucial to understand unique biology and histology of CRCLM. CRCLM grow mainly following three histologic growth patterns (HGPs), extensively described in Latacz et al.^7^: desmoplastic or encapsulating (dHGP), pushing, and replacement (rHGP), the latter two often classified as nondesmoplastic (non‐dHGP). These HGPs display differential histological characteristics where the TME plays a key structural role. The desmoplastic or encapsulating HGP is characterized by a fibrotic rim separating the tumor from the hepatocytes, relying on angiogenesis for blood supply.^8^ The rim comprises carcinoma‐associated fibroblasts (CAFs), extracellular matrix proteins, and a dense lympho‐myelocytic infiltrate, usually found in the outer portion of the rim. In approximately the 20% of cases, the fibrotic capsule forms the complete tumor–host interface (THI), associated with favorable prognoses in large patient cohorts.^7^ rHGP is characterized by hepatocyte–tumor cell contact, defining the THI as the invasive front in deep contact with liver parenchyma.^8^ It is the most common pattern, present in at least a portion of the THI in 75% of patients. Vessel co‐option is the preferential form of blood supply. Lesser frequent is pushing HGP, accounting for only 2–3% of cases.^8^ It is characterized by hepatocyte flattening at the THI due to expansive tumor growth.

The current failure of immunotherapy strategies in CRCLM, particularly for MSS tumors, highlights the need to expand our understanding of the TME biology. While some mechanisms of cytotoxic cell siphoning by certain macrophage subpopulations are known,^9^ the intricate heterotypic interactions between the tumor–stroma require in‐depth characterization. Likewise, the HGPs and their inherent biology could serve as a predictive tool for more tailored treatment strategies based on the HGP.^10^

Thus, this study aims to characterize the TME in each HGP by quantifying different cell types, including lymphoid, myeloid, and CAFs, using a multiplex antibody‐based platform in a tissue microarray (TMA) of 100 patients. Our results suggest that patients could benefit from more effective and personalized preoperative treatment depending on the HGP of their metastases. The different biology of HGPs highlights the need to be able to predict these patterns, which our group^11^ and others are working on.^12^

## 2 RESULTS

### 2.1 Validation of the HGP prognostic value

The prognostic value of HGP patterns is widely demonstrated by different series and different research groups.^13^, ^14^, ^15^, ^16^ We have validated this prognostic value to corroborate that the results that we present below fall within the usual parameters of research on HGPs.

Survival analyses in the entire consecutive cohort of 135 patients indicate better outcomes for encapsulated metastases compared with non‐dHGP metastases (Cox *p* value 0.016; HR = 2.83, 95%CI 1.21–6.59; Figure S1). dHGP metastases were smaller than nondesmoplastic metastases (*t*‐test, *p* = 0.006; Figure S1). Table S1 illustrates the clinical associations of the samples with the HGPs.

### 2.2 Lymphoid subsets assessment in CRCLM

**FIGURE 1:**
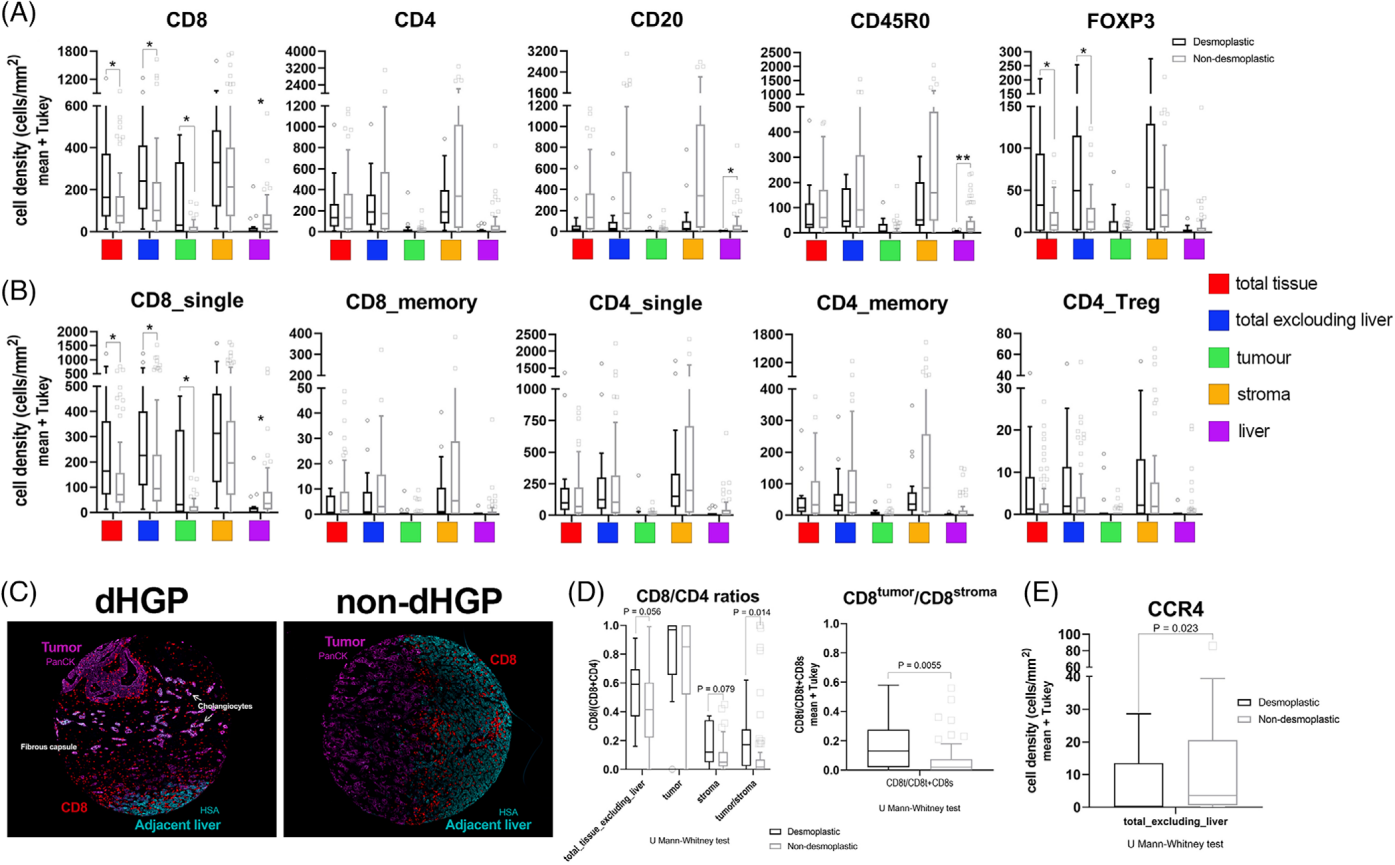
Characterization of lymphoid cells and subsets in desmoplastic and nondesmoplastic liver metastases. (A) Box‐plot graphs (mean values and interquartile range) of cell densities (cells/mm^2^) for the different lymphoid cell markers used and measured in the different compartments assessed. To compare differences, we used Mann–Whitney *U* test with a Pratt correction for zeros and ties plus Bonferroni adjustment for multiple testing. Asterisks denoted statistical significance, *p < 0.05. (B) As above, box‐plot graphs for main lymphoid subsets. (C) Representative images of CD8 staining for an encapsulating metastasis (dHGP; left image) and nondesmoplastic metastasis (non‐dHGP, right image). As shown in the left image, CD8 cells were more abundant on the dHGP metastasis, both in stromal regions, basically in the fibrous capsule but also inside the tumoral compartment, allocated over the tumor cells. On the contrary, on the right image (non‐dHGP metastasis), CD8 were mainly retained in the liver and in the tumor liver interface (TLI). As segmentation markers, PanCK, in pink, for Pan‐Cytokeratin, and HSA, in cyan, for hepatic‐specific antigen. (D) CD8‐to‐CD4 ratios in the different compartments (left panel). In addition, we computed the ratio CD8^tumor^/CD8^stroma^ (right panel), a metric that estimates the tumor exclusion of the CD8 infiltrates, being higher in the non‐dHGP metastases (*p* = 0.0055; Mann–Whitney *U* test with Pratt correction for ties). (E) We assessed the protein expression of CCR4, a cytokine receptor present in Th2 lymphocytes. Nonencapsulating metastases displayed higher densities of CCR4^+^ cells in the tumoral areas of the TMA cores (tumor cells + stromal areas; *p* = 0.023, Mann–Whitney *U* test with correction for ties). CCR4 was measured by conventional IHC.

When looking at lymphocyte subsets, the striking differences were observed concerning CD8^+^ T‐cells. This analysis showed higher infiltration in dHGP of CD8^+^ cells when assessing the whole TMA core (total_tissue, adj *p* = 0.045), as well as excluding the adjacent liver (adj *p* = 0.045) and over the tumor nests (adj *p* = 0.045), suggesting that the differences observed in the area of whole TMA dHGP cores were attributable to higher infiltration inside the malignant lesion, particularly in the tumor compartment since the stromal areas did not displayed differences between HGPs (Figure 1). The higher infiltration by CD8^+^ cells was further confirmed in the second cohort, for both the invasive margin (*p* < 0.0001) and central areas of the specimen (*p* < 0.0001; Figure S2). Interestingly, the adjacent liver parenchyma in non‐dHGP metastases displayed higher CD8 infiltration, although density values were low compared with the other compartments (adj *p* = 0.045; Figure 1 and Table S2). Noticeably, there were no differences between HGPs regarding the MSI/MSS status; both patterns displayed around 10% of cases with MSI (Table S3). Neither CD4, CD45R0 nor CD20 provided differences between HGPs regarding the different intratumoral compartments, although all density values were higher in non‐dHGP. Values did not reach statistical significance, with the exception for CD45R0 (adj *p* = 0.001) and CD20 (adj *p* = 0.012), higher in the adjacent liver of non‐dHGP metastases.

Surprisingly, FoxP3 was found at higher densities in dHGP metastases, despite its typical association with Treg lymphocytes when combined with CD4 or CD8. Nuclear FoxP3 staining was also detected in tumor cells, hepatocytes, and CD45R0 cells (Figure S2). The significance of nuclear FoxP3 expression in tumor cells and hepatocytes is unclear. However, it has been associated with improved outcomes in hepatocarcinoma^17^ and breast cancer.^18^

Next, we identified common subpopulations using the evaluated markers. Figure 1 illustrates that there were no observed differences between HGPs in CD4^+^_single, CD4^+^CD45R0^+^ (CD4_memory), or CD4^+^FoxP3^+^ (CD4_Tregs). Notably, we found relevant results regarding CD8^+^_single cells, particularly within tumor cells (intraepithelial; adj *p* = 0.038; Figure 1). These findings potentially suggest that CD8^+^_single cells in dHGP reach the tumor cells and interact with them. Furthermore, CD8^+^_single cells exhibited higher densities in the adjacent normal non‐dHGP liver parenchyma, indicating that cytotoxic cells could be retained in the peritumoral zone, without the ability to reach malignant cells. Metastases presented similar values of retained infiltrates in the stroma regardless of the histological pattern. Regarding the CD8‐to‐CD4 ratio, dHGP patients displayed higher intraepithelial‐CD8‐to‐stromal‐CD4 (adj *p* = 0.014) suggesting increased cytotoxic activity in encapsulated metastases. This is further supported by a higher intraepithelial‐CD8‐to‐stromal‐CD8 ratio as well in encapsulated metastases (adj *p* = 0.0056; Figure 1D).

Given the aforementioned results, particularly the low intraepithelial‐CD8‐to‐stromal‐CD4 ratios in the non‐dHGP metastases and the reduced presence of CD4_Tregs, we explored other protumoral T‐helper populations influenced by HGPs. Among CD4^+^ T‐helper subsets, Th2‐cells are present in many different tumors.^19^ These cells are described to drive the macrophage polarization toward M2‐type, contributing to an immunosuppressive TME.^20^ To evaluate the contribution of Th2 lymphocytes and their distribution in the different HGPs, we decided to evaluate the CCR4 receptor (also known as CD194). The staining data revealed a significant increase in CCR4^+^ cells in non‐dHGP (Figure 1), suggesting their potential role in creating an immunosuppressive environment in non‐dHGP metastases.

Summarizing, encapsulating metastases display a greater infiltration, although with discrete values, of cytotoxic cells on tumor cells than nonencapsulating metastases, a fact that is confirmed by a higher intraepithelial CD8‐to‐stromal CD4 ratio.

### 2.3 Myeloid cells are more abundant in the stroma of non‐dHGP metastases

Regarding myeloid cells, we stained the total macrophage population with CD68, M1‐macrophages (CD68^+^CD163^−^cells) and M2‐macrophages (CD68^+^CD163^+^cells). The other assessed myeloid markers (CD163^+^_single, Calprotectin) and their combinations are difficult to associate to one particular subset of cells because of the promiscuity of these markers in the myeloid lineage. Consequently, it may be more appropriate to consider these markers from a functional perspective, recognizing them as part of an immunosuppressive population.^21^, ^22^

**FIGURE 2:**
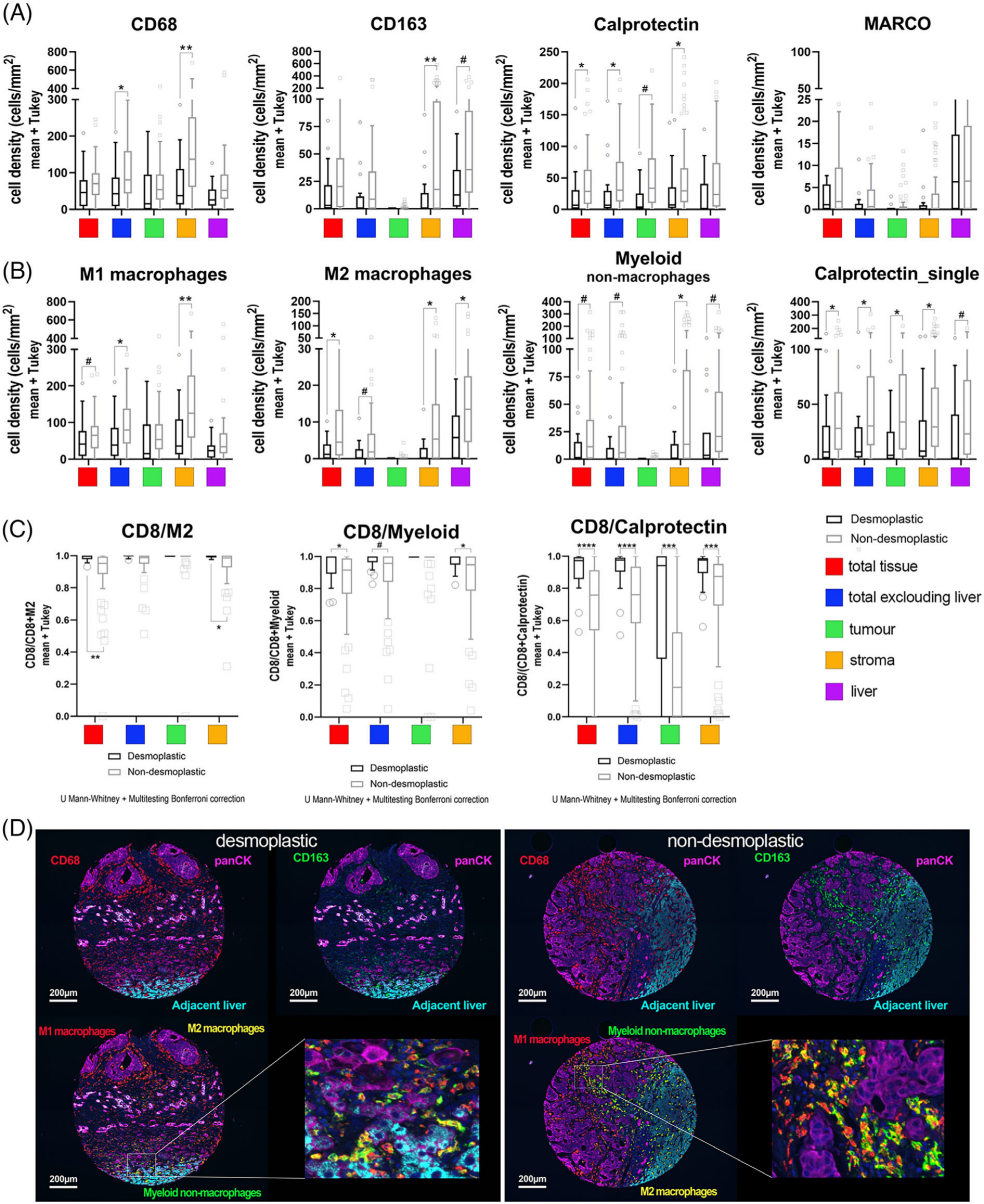
Characterization of myeloid cells and subsets in desmoplastic and nondesmoplastic liver metastases. (A) Box‐plot graphs (mean values and interquartile range) of cell densities (cells/mm^2^) for the different macrophage and myeloid cell markers used and measured in the different compartments assessed. Compartments were determined using morphology and segmentation markers (PanCK, hepatic‐specific antigen, and nuclei staining). (B) As above, box‐plot graphs for polarized macrophages, myeloid nonmacrophages (CD68^−^CD163^+^) and Calprotectin_single cells. (C) SIA, signature of immune activation, ratio of CD8 cells to different immunosuppressive myeloid cells, either M2 macrophages, myeloid nonmacrophage, or Calprotectin^+^ cells. These metrics are considered a surrogate marker for the antitumoral status versus the immunesuppressive environment. (D) representative images of the different macrophage populations assessed. Left panel, for desmoplastic metastases and right panel for nondesmoplastic metastases. To compare differences in A, B, and C, we used Mann–Whitney *U* test with the Pratt correction for zeros and ties plus Bonferroni and/or false discovery rate adjustment for multiple testing. When Bonferroni adjustment was to astringent we applied FDR for multitesting correction. Asterisks denoted statistical significance, *p < 0.05; **p < 0.001; ***p < 0.0001 after Bonferroni correction. # denoted FDR < 0.05.

First, we explored the total values of the markers used. With the exception of MARCO, all the myelophagocytic markers were displayed at higher densities in the stroma of the non‐dHGP (CD68^+^ adj *p* = 0.002; CD163^+^ adj *p* = 0.02; Calprotectin^+^ adj *p* = 0.020; Figure 2).

Regarding the association of these markers with known cell subsets, non‐dHGP metastases displayed higher densities of M1 and M2‐macrophages in their stroma (adj *p* = 0.002 and adj *p* = 0.022, respectively).

In addition, we observed a high density of CD68^−^CD163^+^cells, that we named myeloid nonmacrophage cells,^23^ in the stroma (adj *p* = 0.022) and normal adjacent liver (a trend for Bonferroni test, adj *p* = 0.057; FDR adj *p* = 0.028).

Calprotectin is a marker associated with neutrophils, monocytes, inflammatory macrophages, and other myeloid cells.^24^ Recently, the presence of Calprotectin^+^ macrophages, presumably derived from MDSC (myeloid‐derived suppressor cells), has been associated with poor responses to immunotherapy.^25^ Our data seem to indicate that most of the Calprotectin^+^ cells do not express the other macrophage markers like CD68 and CD163, at least in intratumoral areas (Figure S3). However, most of the Calprotectin^+^ cells also express Myeloperoxidase and CD15 (Figure S4), common markers for all the cells of the myeloid lineage. Interestingly, Calprotectin_single cells are statistically more abundant in non‐dHGP metastases in all the compartments assessed (Table S2). From the information provided by the combination of antibodies used in the panel of myeloid cells, we cannot firmly conclude that the Calprotectin_single cells correspond to neutrophils. However, we illustrated that they do not coexpress either CD68 or CD163. However, the complementary stains performed with the Calprotectin/CD15/Myeloperoxidase triple labeling seem to indicate that they indeed mostly correspond to neutrophils (Figure S4). In addition, Calprotectin^+^ cells, both in intratumoral areas and adjacent liver showed two different morphologies, some round cells, the majority of them (CD163^−^), and another small set of cells with spiculated morphology, that seems to be coexpressing also CD163 (Figures S3 and S5).

To complement these results, we assessed Myeloperoxidase by conventional IHC staining. In the same way as for Calprotectin, nonencapsulating metastases had a greater infiltration by cells Myeloperoxidase^+^ that was mostly absent in dHGP metastases (*p* < 0.0001; Mann–Whitney *U* test; Figure S6).

Both Calprotectin and Myeloperoxidase were also validated in the second cohort, where Calprotectin was present at higher densities both in the invasive margin and central areas of the nondesmoplastic metastases (*p* = 0.0002 and *p* = 0.0128 respectively; Figure S6), as well as Myeloperoxidase (mostly absent, both in the invasive margin and central areas and mainly retained in the outer part of the capsule; *p* = 0.0009 for the invasive margin and *p* = 0.033 for central areas).

Interesting results have been obtained for MARCO. While we did not see differences regarding the HGPs, no MARCO^+^ cells have been observed inside the tumor mass. This result was further validated using conventional IHC in a different cohort of patients as well as using CD5L another specific marker for Kupffer cells (Figure S5). Although some recent publications have been evidenced different macrophage populations expressing MARCO and CD5L in liver metastases at the RNA level, we hypothesize that either these two scavenger receptors are regulated at the translational level once Kupffer cells are recruited into the tumor,^26^ or Kupffer cells do not infiltrate tumors. Likewise, as we detailed in the Figure S5, while in normal liver parenchyma the Kupffer cells coexpress both MARCO and CD163, the closer the cells to the tumor, near the THI, the lesser the MARCO staining, being completely negative inside the tumor, thus, supporting our first hypothesis.

As a summary, nonencapsulating metastases showed greater infiltration of myeloid cells, regardless of cell subtype.

### 2.4 Ratio CD8 and myeloid cells, a value to measure the activation status of the immune system

The relative density of CD8^+^ cells to the specific subset of M2‐macrophages provided a metric that has been considered a balance between anti‐ and protumoral immunity, which has been named SIA, for Signature of Immune Activation.^27^ In the same vein, given the high abundance of myeloid nonmacrophage cells and Calprotectin^+^ cells in CRCLM we also assessed the ratio CD8^+^‐to‐myeloid nonmacrophage cells and CD8^+^‐to‐Calprotectin. Lower values of these three metrics mean higher immunosuppressive capacity. Thus, as shown in Figure 2, compared with non‐dHGP, dHGP metastases displayed statistically significant higher values (adjusted *p* < 0.05) for all of the three parameters, particularly in the stroma compartment, and even in the tumor compartment for the CD8^+^/Calprotectin^+^ ratio, reinforcing the concept that nondesmoplastic metastases displayed a potent immunosuppressive environment.

To further assess the immunosuppressive microenvironment we evaluated the phosphorylation of SMAD2, as a surrogate marker of the activation of the TGFβ pathway. Our results, summarized on Figure S6 show that pSMAD2 was significantly more present on non‐dHGP CRCLM (*p* = 0.0026; Mann–Whitney *U* test), both in both tumor cells and stromal cells, while 10 out of 22 dHGP metastases were completely negative for pSMAD2.

Recapitulating, nonencapsulating metastases present greater stromal immunosuppressive activity based on the greater presence of myeloid cells and greater phosphorylation of SMAD2 in both the stroma and the tumor.

### 2.5 Unexpected prognostic value of Calprotectin

**FIGURE 3:**
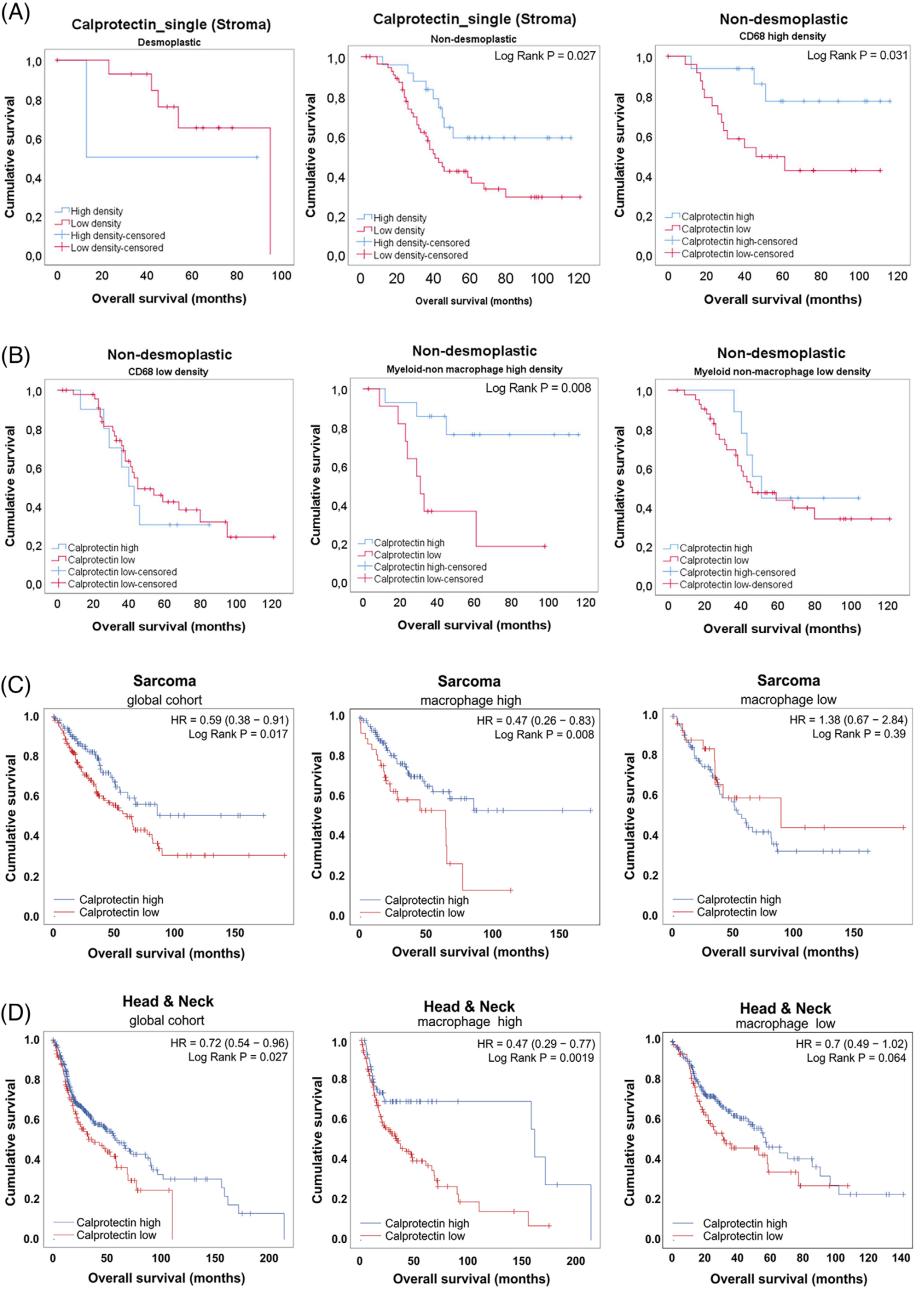
Kaplan–Meier survival analyses: (A) different behavior of Calprotectin_single enriched samples (Stroma) between encapsulated liver metastases (desmoplastic HGP; left panel) and nonencapsulated liver metastases (nondesmoplastic HGP, middle panel). However, when taking into consideration nondesmoplastic metastases, the high density of Calprotectin was associated with good prognosis only in a subset of patients enriched in macrophages (Log Rank *p* = 0.031; A, right panel) or also enriched in myeloid nonmacrophage cells (B, middle panel; Log Rank *p* = 0.008). In low‐enriched macrophages or myeloid cells metastases, Calprotectin_high cases did not confer better outcome (B, left and right panels). We used the mean density value for CD68 or myeloid nonmacrophage cells as a cut off in the entire cohort of metastases. Similar results have been obtained using third‐party data in sarcoma (C; left panel) and in head and neck cancer (D, left panel) high expression values of Calprotectin (considered here as the mean expression of S100A8 and S100A9 genes obtained in RNAseq public databases) provided a better prognosis than low expression in tumors enriched in macrophages.

Calprotectin is an heterodimer formed by proteins S100A8 and S100A9 has been associated with poor outcome in different tumoral settings.^28^, ^29^ However, when we assessed the prognostic value of this marker, astonishingly, we observed a difference between HGPs. As detailed in Figure 3, low density of Calprotectin^+^ cells was associated with better prognosis in desmoplastic metastases. On the contrary, high‐density values of Calprotectin^+^ was associated with better outcome in nondesmoplastic metastases, both using the mean density as a cut‐off (Figure 3; Log Rank *p* = 0.027) or tertiles (data not shown). Interestingly, this unexpected result in nondesmoplastic metastases occurred exclusively in those nonencapsulated metastases that had a high density of other types of myeloid cells, such as macrophages and nonmacrophage myeloid cells, identifying a subgroup of patients with nondesmoplastic metastases with a much more favorable prognosis (Figure 3). Similar results have been evidenced in public series of head and neck tumors and sarcomas, where Calprotectin also seems to confer a better prognosis in samples with an enrichment in macrophages.^30^

These results might indicate different Calprotectin_single^+^ cells between encapsulating and nonencapsulating metastases and highlights that a deeper characterization of Calprotectin‐expressing cells is needed.

### 2.6 Distinct HGP metastases have different CAF subsets repertoire

**FIGURE 4:**
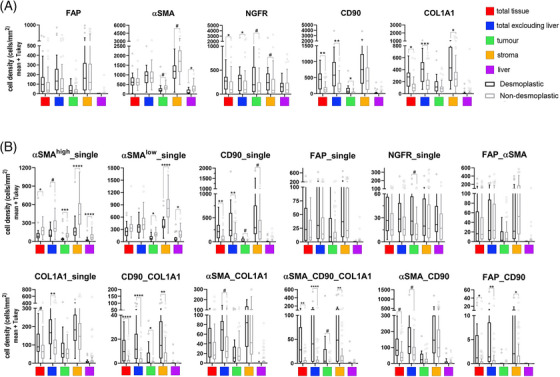
Characterization of carcinoma‐associated fibroblasts and subsets in desmoplastic and nondesmoplastic liver metastases. (A) Box‐Plot graphs (mean values and interquartile range) of cell densities (cells/mm^2^) for the different CAFs cell markers used and measured in the different compartments assessed. Compartments were determined using morphology and segmentation markers (PanCK, hepatic‐specific antigen, and nuclei staining). (B) Among all the possible combinations of markers used to characterize the CAFs, we selected those that had a representation of at least a fraction equal to or greater than 15% of the total fibroblasts in any of the samples. The densities of the twelve selected were further assessed in the different compartments. To compare differences, we used Mann–Whitney *U* test with the Pratt correction for zeros and ties plus Bonferroni and/or false discovery rate adjustment for multiple testing. When Bonferroni adjustment was to astringent we applied FDR for multitesting correction. Asterisks denoted statistical significance, *p < 0.05; **p < 0.001; ***p < 0.0001 after Bonferroni correction. # denoted FDR < 0.05.

To characterize whether metastases with different HGPs may have different CAF subsets, we stained TMA samples with classical CAF markers, FAP and αSMA, as well as with CD90 and NGFR, proteins characteristic of the main CAF precursors in liver, portal fibroblasts (PF), and hepatic stellate cells, respectively. We also included Collagen 1 in the panel, given the role as a possible restraining‐CAF marker according to different recent publications.^31^, ^32^ We performed a first analysis assessing the total cell densities for each marker in the different compartments considered, the results of which are shown in Figure 4 and Table S2. It is important to note that the metric used throughout the article is cell density (cells per area unit for each of the segmented compartments). And it is especially notable for mesenchymal cells, since, although metastases with dHGP present larger areas of stromal tissue, this fact does not always have an impact on greater cell density. With this initial analysis we saw that there were few differences between HGP with respect to the classic CAF markers αSMA and FAP (Figure 4 and Figure S7; Table S2). In relation to CD90 and NGFR, there are significant differences between HGPs in relation to CD90, taking into account all the tissue available (total punch) as well as considering exclusively the tumoral part (adj *p* < 0.001) and the tumor compartment (adj *p* = 0.018). This could be due to the degree of glandular differentiation, well differentiated for dHGP and poorly differentiated for non‐dHGP. Well‐differentiated glands usually present a fine line of myofibroblasts giving support and shape to the gland in its most distal part. Regarding NGFR, the differences were more discrete between HGP, although dHGP CRCLM did present a higher density considering all the core tissue (adj *p* = 0.031) and excluding adjacent normal liver tissue (adj *p* = 0.031). Interestingly, COL1A1, a classical myCAF (myofibroblastic CAF) marker, recently being associated with a subset of rCAF (restraining CAFs^31^, ^32^) was present at higher density in encapsulating metastases, both considering the whole tumoral area (excluding the adjacent liver; adj *p* < 0.0005) and particularly in the stromal compartment (adj *p* = 0.05; Figure S8).

Having initially considered the antibodies used individually, we next wanted to evaluate different subpopulations of CAFs based on the combination of different antibodies, as well as the fluorescence emission intensity. First, regarding αSMA, myCAFs are described elsewhere as displaying high expression of αSMA, while iCAFs (inflammatory CAFs) express also αSMA but at lower levels.^33^, ^34^, ^35^ We used the mean intensity value detected for αSMA as a cut‐off to discriminate between αSMA^high^ and αSMA^low^ cells. Then, we combined the different antibodies in order to define populations of CAFs that could be associated with their functionality. To select CAF subsets, we considered all possible combinations (Figure S9), and we discarded those present at very low fractions in the entire cohort and selected for further analyses those subsets with at least fraction above 15% in any of the samples, twelve different subsets (Figure 4). Thus, αSMA^high^_single was significantly present at higher densities in all the different spatial compartments of non‐dHGP CRCLM, even after multiple testing adjustment. We manually excluded tubular structures from the analysis to avoid αSMA staining of pericytes.

**FIGURE 5:**
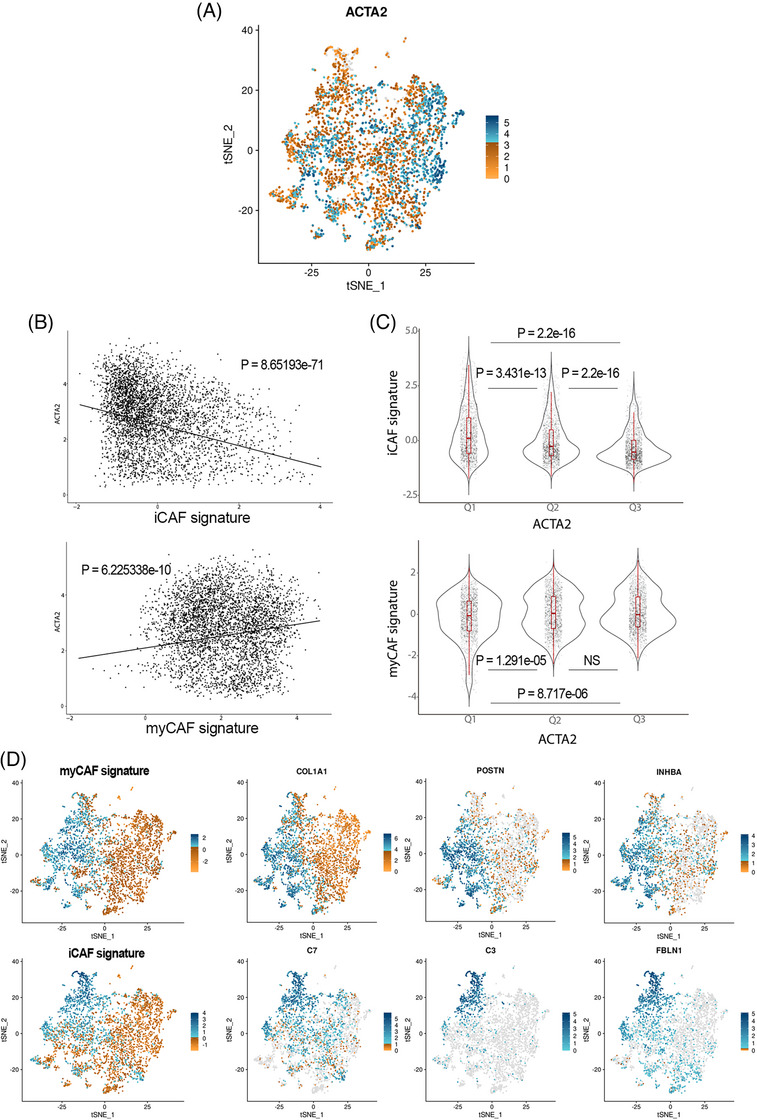
αSMA^low^_single cells express high values of a iCAF signature. We used single cell transcriptomic data from 4397 CAFs isolated from six different colorectal cancer metastases. We visualized the tSNE plot for ACTA2 expression (A), showing a wide range of gene expression among all the CAFs. Thus, we computed a ssGSEA score for an iCAF and myCAF signatures (Supporting Information) and calculated the correlation of these ssGSEA scores with the ACTA2 expression. As shown in (B) (top panel), the inverse correlation between ACTA2 and the iCAF scores indicates that CAFs with high ACTA2 expression displayed low iCAF signature values (Spearmen correlation *p* = 8.65e−71) and just the opposite for a myCAF signature (bottom panel). When we separated the expression of ACTA2 into tertiles, the first tertile (lowest expression of ACTA2) was the one with the highest expression of the iCAF signature in a statistically significant way in relation to the other two tertiles. Finally, we depicted in (D) the tSNE plots for the iCAF and myCAF ssGSEA scores for the 4397 CAFs and the expression of some iCAF and myCAF classical genes. In gray, we represented values equal to zero, in orange scale the nonzero values up to percentile p66 and in blue scale the values above percentile p66.

To address the subset of iCAFs, associated with protumoral functions,^36^ our panel, by technique limitations (number of markers simultaneously assessed), did not included an specific iCAF marker as IL6, HGF, CCL2 or coagulation Factor D, in any case, proteins difficult to determine correctly by means of immunohistochemical techniques. We rely on the assessment of αSMA^low^_single (thus, negative for all other markers). To further confirm the association of αSMA^low^ CAFs with the population of inflammatory CAFs,^33^, ^34^, ^35^ we used scRNAseq data from Giguelay et al.^37^ (GEO reference GSE158692). As illustrated in Figure 5, the expression of the gene ACTA2 is highly heterogeneous among the 4397 analyzed CAFs (Figure 5). Likewise, the expression of ACTA2 shows an inverse correlation with the ssGSEA values for an iCAF signature (Spearman correlation *p* = 8.65e−71) and it is clearly observed that the CAFs with the lowest expression of ACTA2 (first tertile) are those with the highest expression of the iCAF signature (Figure 5). Once corroborated the association of the expression of ACTA2 with the iCAF signature, we considered αSMA^low^ CAFs with the iCAF phenotype. Therefore, αSMA^low^_single cells were present at higher densities in nondesmoplastic metastases, both in the stroma compartment (adj *p* = 0.000041) as well as in the thin stromal septae encompassed within the tumor compartment (adj *p* = 0.0033).

Regarding the different subsets that express COL1A1, COL1A1_single, CD90_COL1A1, αSMA_COL1A1, and αSMA_CD90_COL1A1 were mostly detected in encapsulated metastases, suggesting this role of restraining the tumor progression already described in aforementioned publications.^33^, ^34^, ^35^ All these subsets might be defining the ECM–CAF subset associated with restraining functionalities. Interestingly, as displayed in Figure S10, these COL1A1 subsets do not coexpress neither FAP, Periostin, and other ECM markers, thus suggesting different ECM–myCAFs. These other myCAFs are probably represented by FAP expressing subsets, either FAP_single, FAP_αSMA without differences regarding HGP or FAP_CD90, the latter statistically more present in dHGP although at very low densities. FAP has frequently been described as a myCAF marker associated with protumoral functions such as immunosuppression.^38^ Therefore, we postulated that CAFs positive for FAP in combination with any other marker but negative for COL1A1 might be a tumor‐promoting myCAF population.

Thus, encapsulated metastases are enriched in Collagen 1‐producing mesenchymal cells, particularly on the fibrotic rim, while nonencapsulated metastases displayed higher densities of protumoral FAP^+^ CAFs as well as inflammatory CAFs.

### 2.7 Interrelationship between different cells of the TME

**FIGURE 6:**
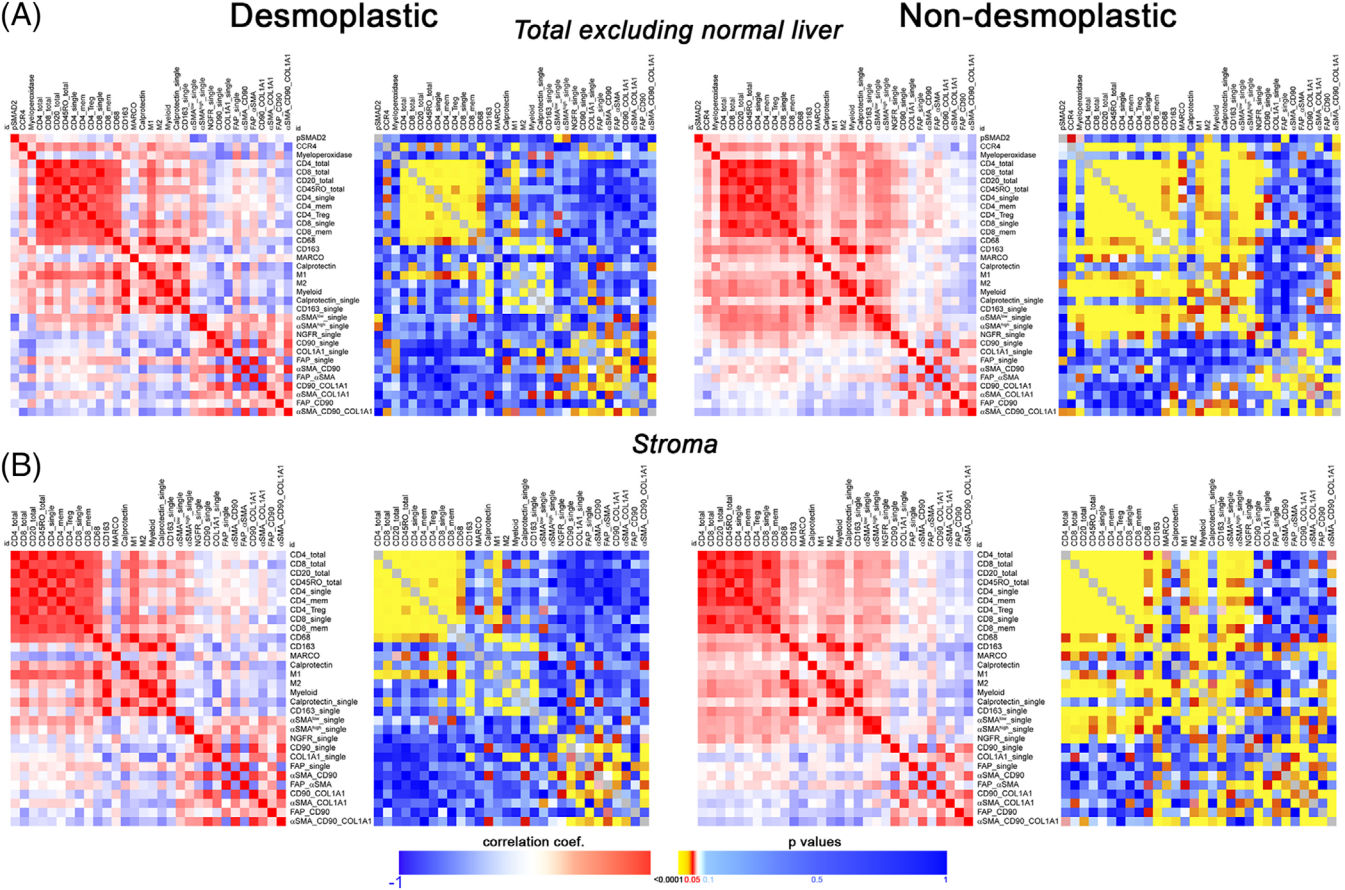
Spearman correlation analysis (blue‐to‐red heatmaps; bar scale −1 to 1 illustrates correlation coefficients) in dHGP and non‐dHGP CRCLM considering the cell subsets evaluated in both total tissue excluding liver (A) or in the stroma only (B). Corresponding yellow‐to‐blue heatmaps illustrate the *p* values for each Spearman correlation. Heatmaps were made using the website https://software.broadinstitute.org/morpheus/.

We aimed to explore whether particular cell subsets might be infiltrating in a coordinated fashion. To delve into this possibility, we performed a Spearman correlation analyses, both for the total tissue available excluding the normal adjacent liver or considering the stroma compartment only (*p* values and correlation coefficients are depicted on Table S4. As illustrated in Figure 6, both for desmoplastic and nondesmoplastic metastases, lymphoid cells tend to occur together and most of these cell correlations were statistically significant (*p* < 0.05). Of note, the high lymphocytic infiltration was significantly correlated with CD163^+^ cells as well as with M2‐macrophages and nonmacrophage myeloid cells in the case of nondesmoplastic metastases, suggesting a coordinated infiltration, unlike the encapsulated metastases. Particularly for CD8_total and CD8_single, there is a statistically significant correlation with CD163^+^, M2‐macrophages and myeloid cells (but not for calprotectin), which could be indicative of an immunosuppressive siphoning of cytotoxic cells in nondesmoplastic metastases, the opposite occurring in encapsulated ones (Figure 6).

Regarding CAFs, the abundance of αSMA^low^_single subset was positively correlated with different lymphoid and myeloid subsets in nondesmoplastic metastases not so in encapsulated metastases.

## 3 DISCUSSION

The HGPs observed in CRCLM have intrigued pathologists for years.^39^, ^40^ During early 2000s, several studies explored their potential connection to patient prognoses.^15^ These studies also assessed the biological features of each HGP.^41^, ^42^, ^43^ Several hypotheses have emerged regarding HGP formation,^44^ although definitive conclusions remain elusive. Predicting the HGP would clinically be valuable,^11^ as some patients may benefit from personalized treatment,^41^ which requires a better knowledge of the particular biology of each HGP as well as their cellular constituents. The TME landscape we observed in CRCLM suggests that HGPs may significantly influence responses to certain therapeutic approaches. Encapsulated lesions exhibit a higher presence of cytotoxic T‐cells in tumor niches, indicating their ability to infiltrate dHGP metastases. In contrast, non‐dHGP metastases not only displayed a lower density of CD8^+^ cells, mostly retained in the liver, and higher values of CCR4^+^ Th2‐cells, but also greater levels of different myeloid cell. This fact, along with higher pSMAD2 expression in both tumor and stromal cells, creates a more immunosuppressive scenario compared with encapsulated metastases. Since non‐dHGP accounts for approximately 80% of the cases, these findings may explain the failure of immunotherapy in most patients with CRCLM.^9^, ^45^ Conversely, our results indicated no significant differences in any assessed compartments concerning CD8 memory cells (CD8^+^CD45R0^+^). Several memory T‐cell subsets were defined, including stem‐like, effector memory, central, and tissue resident memory T‐cells.^46^ Notably, resident memory T‐cells have been associated with better clinical outcomes in various cancer types.^47^, ^48^, ^49^, ^50^ Although effector T‐cells have long been recognized as important antitumor protection mediators, efficient immunity against cancer may require long‐lived T‐cell immunity due to the chronic nature of this disease.^51^ Therefore, one might expect higher levels of CD8 memory cells in dHGP. However, it is known that the functional state of CD8 T‐cell subsets may differ for specific pathogens and tumor types,^52^ suggesting that antitumor immune responses vary in each tumor context. Consistent with our results, in CRC, other authors also reported higher levels of CD8 cells, rather than memory cells, infiltrating the tumor compartment.^23^ This suggests that CD8 cells negative for CD45RO contribute to the immune response against tumors in the context of CRC, whether primary or metastatic. Additionally, the ratios CD8^tumor^/CD8^stroma^ and CD8^tumor^/CD4^stroma^ indicated a more proficient immunosuppressive environment in non‐dHGP.

It has been extensively described that the presence of tumor‐associated macrophages and other myeloid cells is associated with poor prognosis in solid tumors.^53^ These cells can enhance tumor progression, promoting immunosuppression, angiogenesis, inflammation, and chemoresistance.^54^ Particularly in the hepatic context, the liver has the largest density of CD68^+^ macrophages of the entire body, mainly two big populations, inflammatory macrophages, expressing at the RNA level S100A8/S100A9, CXCL8 among others genes, and noninflammatory macrophages expressing MARCO, CD5L, CD163, the latter considered as Kupffer cells.^26^ In CRCLM, we observed higher amounts of all assessed myeloid cells in non‐dHGP, even those categorized as M1‐macrophages (CD68^+^CD163^−^), in fact displayed at higher densities than M2‐macrophages, as reported previously.^23^ Although we did not assess an specific M1 marker as CD80 or CD86, we cannot exclude the presence of M2‐macrophages expressing CD68^+^CD163^−^ and CD206^+^, combination not tested in our study. Therefore, higher densities of M2‐macrophages and nonmacrophage myeloid cells, correlation of these myeloid cells with subpopulations of lymphoid cells, consistent with lower ratios of CD8 to these cells, suggest that the siphoning phenomenon already described occurs in these nonencapsulated metastases.^9^

In our samples we observed a higher density of both Calprotectin^+^ and Calprotectin^+^_single cells in nonencapsulated metastases. As mentioned before, Calprotectin is an intracellular calcium‐binding protein expressed by different cell types, mainly neutrophils, monocytes and MDSCs. It is induced by several soluble factors, as TNFα, IL1α and β, LPS, IL10, VEGFα, TGFβ, among others. Interestingly, the induction by both proinflammatory and anti‐inflammatory stimuli is cell‐type dependent.^55^ In most clinical cancer scenarios, Calprotectin is conferring a bad outcome.^28^ However, there are evidences suggesting that the function of this protein can be diverse.^56^ The reasons are not fully elucidated, but may stem from the TME complexity. Notably, in nondesmoplastic metastases with high densities of other myeloid cells, Calprotectin appears to be associated with better outcomes, outlining a subgroup of patients with nondesmoplastic metastases with very favorable prognosis. Similar results have been obtained from public databases of sarcoma and head and neck cancer cohorts. Although results seem to be consistent, there is no clear explanation yet. Some authors have suggested that inflammation, both protumoral and antitumoral, may coexist along tumor progression, and the balance between inflammatory forces would dictate the final outcome.^56^

Mihaila et al.^57^ demonstrated that blocking S100A9, one of the two Calprotectin dimers, decreased the secretion of myeloid chemotactic chemokines CCL2, CCL3, and CCL5 in N1 neutrophils. Although further research is needed to understand the crosstalk between neutrophils and other myeloid cells, there is evidence supporting the antitumoral role of neutrophils and their capacity to modulate macrophages and other TME cells.^58^

The complexity is increased by the fact that Calprotectin is expressed by various cell types and at different concentrations.^59^ It is possible that encapsulated metastases have one type of Calprotectin‐positive cells, while nonencapsulated metastases have different subsets, with different functions or capabilities to secrete Calprotectin. Certainly, the considered cell types, including neutrophils, granulocytic‐MDSCs (G‐MDSCs), dendritic cells, monocytes, and others, exhibit overlapping marker expression profiles, making it difficult to precisely discern between them using the current multiplex approach. Thus, it may be more pertinent to delineate them based on their functional roles rather than attributing them to specific cell subsets.

In any case, besides the results in sarcoma and head and neck cancer,^56^ other authors reported associations of S100A9 inflammatory cells with good prognosis in gastric cancer,^60^ as well as therapeutic interventions with recombinant S100A8 in lung cancer.^61^ In CRC S100A8 has been associated with better prognosis, and recombinant S100A8 inhibited both migration and invasion of CRC cells.^62^

Regarding CAFs, we hypothesized that different subsets of these cells exhibit distinct distribution patterns within CRCLM lesions and vary in densities based on the HGP. With the advent of the single‐cell technology, the field of CAFs research has exploded in an endless list of different CAFs subsets, which often appear to overlap probably because an excessive clusterization analyses. Our approach focused on quantifying populations with remarkable density/presence in the CRCLM, neglecting those with very low event numbers, and assigning functions (restraining or protumoral) according to bibliographic data and the known outcomes of the different HGPs.

For FAP we considered two main subsets: fibroblasts negative for all markers except FAP (FAP_single) and a second subset showing αSMA coexpression (both αSMA^high^ and αSMA^low^). A third subset coexpressing CD90 was poorly represented in CRCLM. All other combinations with FAP presented very discrete density values and were not considered. Neither of the two main FAP‐expressing subsets showed differences between HGP's. However, we confirmed that FAP is mainly expressed in the intratumoral stroma of dHGP metastases, not in the peritumoral stroma (capsule). In some cases, we observed an expression gradient from the external portion of the capsule to the internal part in contact with tumor cells.^44^ Other authors have also described that different subsets of CAFs could coexist in the capsule and that they would be functionally grouped forming a gradient from the outer rim in contact with the liver toward the inner rim in contact with the tumor cells.^16^ In fact, it had already been described many years ago that collagen‐secreting cells and cells that produce extracellular matrix modulating proteins were arranged in a zonated manner in the capsule.^63^

FAP has been classically associated with protumoral functions and immunosuppression.^33^, ^38^ In a similar multiplex approach, Pellinen et al.^64^ associated CAF subsets expressing FAP with bad outcome in NSCLC.

Regarding the other classical CAF marker, αSMA, we used a threshold to distinguish between αSMA^high^ (myCAFs) and αSMA^low^ (iCAFs), following the approach by Ohlund et al.,^35^ data validated using a single cell dataset.^37^ Non‐dHGP CRCLM exhibited higher densities of both subsets, which appeared to be positively correlated with different lymphoid and myeloid cells subsets, suggesting a recruiting function of these cells in the stromal compartment. Such correlation was not observed in encapsulated metastases.

One of the most notable differences were observed for Collagen 1, detected at higher densities in different compartments of dHGP‐CRCLM. This marker has been classified as a myCAF marker, and associated with both protumoral and antitumoral functions. Recently, tumor‐derived Collagen I has been associated with protumoral functions, while stromal Collagen I might be acting in an opposite way.^65^ The differences reside in the composition of the protein chains, homotrimers of Collagen 1A1 in the case of tumor‐derived collagen. In the context of CRCLM HGPs, it may be playing a tumor‐restrictive role. Bhattacharjee et al.^31^ previously reported on the antitumor effects of COL1A1 in the context of CRCLMs using mouse models focused on PDAC and CRCLM. They defined two distinct populations of myCAFs: myCAF expressing hyaluronic acid, which were tumor promoting, and myCAF expressing Collagen type I, with tumor‐restraining properties. Akin observation has been reported for cholangiocarcinoma.^66^ Similarly, we define two functionally different myCAF subsets: restraining myCAFs expressing COL1A1 and lacking FAP, and protumoral myCAFs expressing FAP and other ECM proteins like Periostin but negative for COL1A1.

In PDAC, Chen et al.^32^ concluded that, although COL1A1 increases the ECM biophysical stiffness, facilitating abnormal cellular interactions and migration, in balance it has a tumor‐restraining function reducing the proliferation of tumoral cells and increasing the inflammatory response. They observe that COL1A1 deletion in αSMA^+^ CAFs significantly altered the profile of immune cells, increasing the presence of MDSCs and M2‐macrophages, while diminishing T and B cells. Thereby, in PDAC, αSMA^+^COL1A1^+^ CAFs may constitute a tumor‐restricting myofibroblast population. Considering all, we differentiated three CAF subsets expressing COL1A1: αSMA_COL1A1^+^, CD90_COL1A1^+^, and αSMA_CD90_COL1A1^+^ (probably the two latter derived from PF since the CD90 expression). These CAF subsets, present at higher densities in dHGP, would be restraining ECM–myCAF subsets as according to Refs. 31, 32, 66. Probably, the other subsets described are functionally protumoral, mostly expressed at higher densities in nondesmoplastic CRCLM.

**FIGURE 7:**
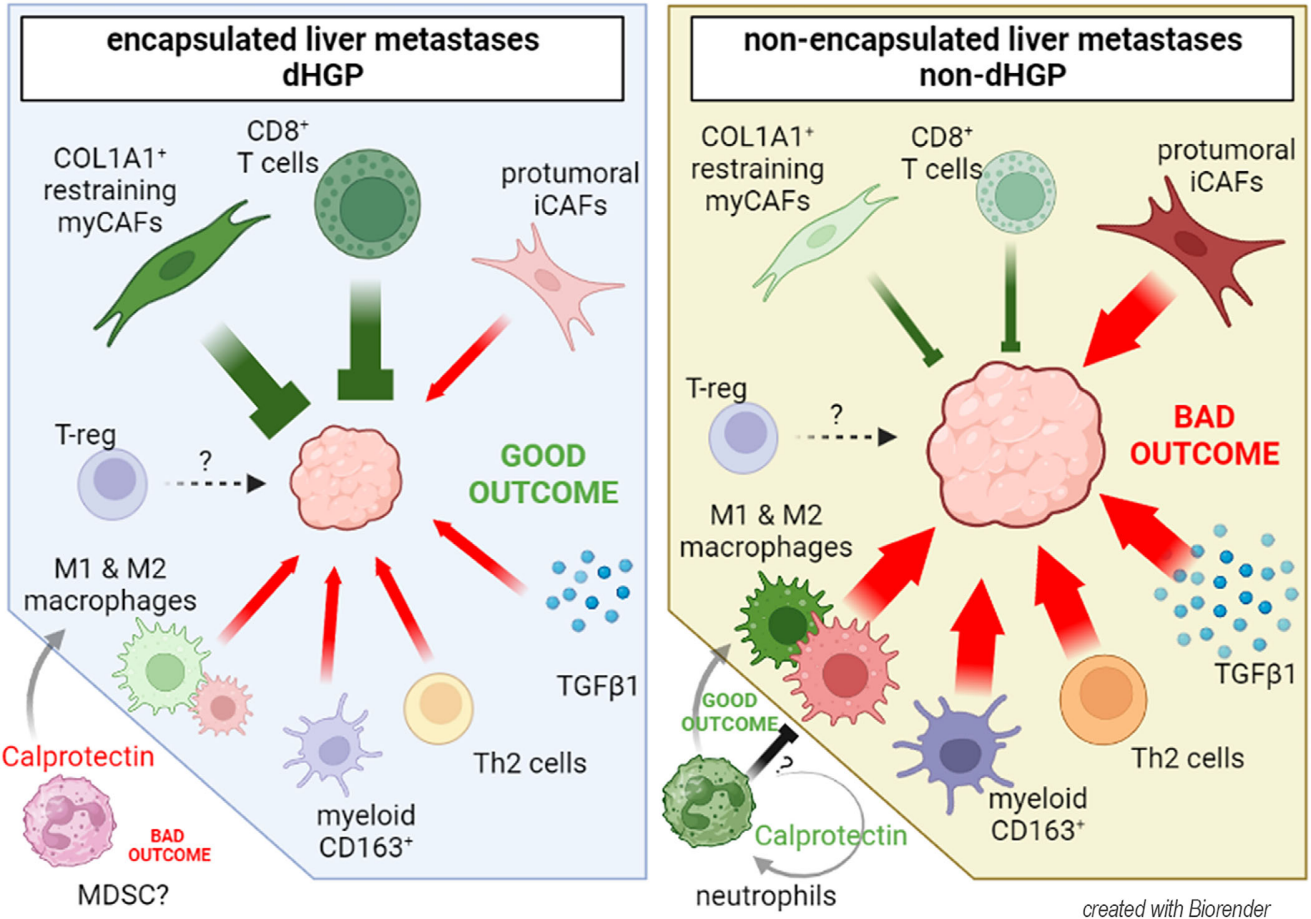
Tumor microenvironment landscapes found in each histologic growth pattern. Two different TME scenarios are depicted depending on the HGP, where most nonencapsulated metastases display a highly immunosuppressive microenvironment while the better outcome of encapsulated metastases might be associated by the presence of cytotoxic T‐cells over the tumor nests and the restraining capabilities of an extracellular matrix enriched in Collagen 1. The dual role of Calprotectin, which seems to have a value dependent on HGP, is noteworthy (created with BioRender).

In conclusion, our results suggest that CRCLM HGPs exhibit distinct TME profiles (Figure 7: Graphical abstract) that may influence treatment responses. Thus, integrating the HGPs into clinical decision‐making is crucial, although currently, they can only be identified post‐surgery. Ongoing studies explore advanced medical imaging techniques, such as computed tomography or magnetic resonance, to develop predictive radiomic signatures. Preliminary results are promising, offering potential for preoperative HGP identification.^67^, ^68^, ^69^ Patients with encapsulated lesions could benefit from antiangiogenic treatments already described^41^ and adoptive T‐cell therapies, since in dHGP CD8^+^ cells can reach tumor niches, less likely to be retained or excluded in the stroma by myeloid cells (siphoning). Conversely, non‐dHGP patients could perhaps be treated with therapies targeting immunosuppressive myeloid cells. Depleting M2‐macrophages and other suppressive myeloid cells would relief the tumor exclusion, restoring the function of the infiltrative T‐cells. Further investigation is needed to better understand Calprotectin's role in CRCLM, especially in relation to HGPs and its interactions within the TME.

## 4 MATERIAL AND METHODS

### 4.1 Patients and TMA building

We obtained retrospective FFPE (formalin‐fixed paraffin embedded) CRCLM samples from surgical specimen from a consecutive cohort (cohort 1) of 135 chemonaive patients managed by the same surgical and pathological team in a single center. Table S5 summarizes the baseline information for this cohort. From the cohort of 135 patients, 100 patients were selected for whom paraffin‐embedded samples were available. The HGP assessment was conducted by trained pathologist (N. R.), who evaluated the entire resected lesion following established guidelines.^7^, ^8^ Only patients demonstrating a pure dHGP—indicated by 100% of the lesion perimeter showing capsule—were categorized as dHGP. Considering that the presence of a small percentage of nondesmoplastic pattern significantly affects prognosis,^15^, ^70^, ^71^ the remaining patients featured lesions showing pure replacement and mixed patterns. Our team's pathologist manually selected invasive margin regions for TMA sections. For patients classified as dHGP, all included areas encompassing liver parenchyma, capsule, and tumor glands. On the other hand, for patients classified as non‐dHGP (pure replacement and mixed patterns), areas exhibiting the replacement pattern were selected, with each core containing both liver parenchyma and tumoral glands. Additionally, two TMA cores were included per patient. For the analysis of the stains, the portal spaces were manually excluded as recommended in the guidelines for assessing the HGPs.^7^, ^8^

We employed a second cohort of 30 patients for validation, comprising untreated CRCLM whole‐slide sections from the same center as cohort 1, with 10 cases classified as dHGP and 20 as non‐dHGP.

### 4.2 Quantitative multiplex staining in TMA sections

Multiplexed immunohistochemical staining was performed with the Opal7 system (Akoya Biosciences). 4 µm thick TMA sections were deparaffinized, rehydrated, and processed for antigen retrieval. The staining procedure involved iterative cycles, applying in each cycle the primary antibody (incubation time 30 min, RT) and the secondary polymerized reporter enzyme staining system (undiluted, incubation time 10 min, RT), and followed by tyramide signal amplification and developing with Opal™ fluorophore (1:100, incubation time 10 min, RT) (Akoya Biosciences). After completing each target, HIAR (microwave, pH6, 15 min) was used to quench endogenous peroxidase activity, facilitate antigen retrieval, and remove antibodies and polymer system from previous cycle. After final staining cycle 4′,6‐diamidino‐2‐phenylindole (DAPI) was applied to nuclear visualization. Details of retrieval buffers, primary antibodies, and amplification systems are described in Supporting Information, Materials and Methods. After staining, slides were scanned using Vectra Polaris. Sample visualization was done using Phenoimager HT System (Akoya) in multiespectral mode at 2 pixels/µm resolution. Multi‐layer images obtained were processed through an spectral algorithm to generate a layer‐composite image. All five filter cubes in the system were used for multispectral imaging, with the saturation protection feature enabled. Signal intensities for each marker were exposure‐normalized, and spectral unmixing was achieved using inForm software

### 4.3 Scanning, visualization, and segmentation of samples

We used PanCK and hepatic‐specific antigen (HSA) for tissue demarcation segmentation. DAPI was used for cell segmentation, which was performed as described in Mezheyeuski et al.^72^ The area at 3 µm (6 pixels) around the nuclear border was considered the cytoplasm area. To stablish the thresholds for each marker positivity, inForm cell phenotyping function was used to choose a representative subset of positive cells as well as a representative negative subset. The training software was performed for each panel of markers separately. Finally, all images were reviewed manually to exclude artifacts, necrotic regions, staining defects, bile ducts, portal tracks, damaged tissue or wrongly segmented areas. Tissue demarcations were designed as follows: total tissue, total tissue excluding normal liver parenchyma, tumor areas, stroma, and normal adjacent liver parenchyma. The expression levels of the markers were used to classify cells into the six main subsets (immune cells, myeloid cells or CAFs, tumor cells, hepatocytes, and marker‐negative cells). Density plots of the protein expression of each marker were used to set the thresholds for “marker‐negative” and “marker‐positive” cells. The number of cells of interest was normalized to mm^2^ (cell density) to minimize variability between TMA cores and between HGPs. Image analysis pipeline is illustrated in Figure S11.

### 4.4 Statistical analyses

For clinical data analysis, overall survival was analyzed through Kaplan–Meier and Cox regression, and Chi‐squared test was used to compare characteristics between dHGP and non‐dHGP. In mIHC TMA analysis, Mann–Whitney *U* test with Pratt correction for ties was applied. Statistical significance was adjusted for multiple testing using Holm‐Bonferroni correction and FDR adjustment, with significance considered at adjusted *p* < 0.05.

## AUTHOR CONTRIBUTIONS

*Investigation; development of methodology; writing first draft; review and editing*: G. G. V. *Data curation; supervision; review and editing*: N. R. *Resources; review and editing*: P. M. *Data curation; supervision; review and editing*: J. C. R. *Data curation; supervision; review and editing*: K. M. *Technical and material support; development of methodology*: M. B. *Investigation¸ review and editing*: N. M. *Bioinformatics; scRNAseq analyses*: M. A. P. *Data curation; supervision; review and editing*: L. L. *Conceptualization¸ investigation; supervision; formal analyses; writing; review and editing*: A. M. *Conceptualization¸ funding; resources¸ investigation; supervision; formal analyses; writing; review and editing*: D. G. M. All authors read and approved the final version.

## CONFLICT OF INTEREST STATEMENT

All authors do not have any financial and personal relationships with other people, institutes, or organizations that could inappropriately influence their work. Additionally, all authors declare that they do not have a close relationship with, or a strong antipathy to, a person whose interests may be affected by publication of the article, an academic link or rivalry with someone whose interests may be affected by publication of the article, membership in a political party or special interest group whose interests may be affected by publication of the article, or a deep personal or religious conviction that may have affected what the author wrote and that readers should be aware of when reading the article. There is no other conflict of interest to disclose.

## ETHICS STATEMENT AND CONSENT TO PARTICIPATE

The study samples were assembled after approval by the Ethics Committee of our institution (IDIBELL; code number PR305/19). Signed, informed consent was obtained from each patient. The study was conducted under the principles of the WMA Declaration of Helsinki.

## Supporting information



Supporting informatin

Supporting information

## Data Availability

The RAW data and InForm files for cell subsets quantification that support the findings of this study are available from the corresponding author on reasonable request.
