# Supplementary material for: The histological growth patterns in liver metastases from colorectal cancer display differences in lymphoid, myeloid, and mesenchymal cells
Source: MedComm (2020). 2024 Nov 19;5(12):e70000. doi: 10.1002/mco2.70000 (PMC11574879; doi:10.1002/mco2.70000)
Supplement: Supplementary file 1 — Supporting informatin [file MCO2-5-e70000-s002.docx]

**Supplementary information**

The histological growth patterns in liver metastases from colorectal cancer display differences in lymphoid, myeloid and mesenchymal cells.

Gemma Garcia-Vicién^1^, Núria Ruiz^1,2^, Patrick Micke^3^, José Carlos Ruffinelli^1,4^, Kristel Mils^1,5^, María Bañuls^6^, Natalia Molina^1^, Miguel A. Pardo^6^, Laura Lladó^1,5^, Artur Mezheyeuski^3,7,8^, David G. Molleví^1,6,8^.

1. Tumoral and stromal chemoresistance group, Oncobell Program, IDIBELL, Gran Via 197-203, L’Hospitalet de Llobregat, Barcelona, Catalonia, Spain.
2. Department of Pathology, Hospital Universitari de Bellvitge, L’Hospitalet de Llobregat, Barcelona, Catalonia, Spain
3. Department of Immunology, Genetics and Pathology, Uppsala University, Uppsala, Sweden.
4. Department of Medical Oncology, Institut Català d’Oncologia, L’Hospitalet de Llobregat, Barcelona, Catalonia, Spain.
5. Department of Surgery, Hospital Universitari de Bellvitge, L’Hospitalet de Llobregat, Barcelona, Catalonia, Spain.
6. Program Against Cancer Therapeutic Resistance (ProCURE), Institut Català d’Oncologia, L’Hospitalet de Llobregat, Barcelona, Catalonia, Spain.

7. Molecular Oncology Group, Vall d'Hebron Institute of Oncology, Barcelona, Catalonia, Spain 8- co-corresponding authors

List of antibodies used in the multiplex panels.

|  | **mIHC Antibodies References** |
| --- | --- |
| **Primary Antibodies** | |
| CD4 | Dako M7319 |
| CD8 | Dako MA5-13473 |
| CD20 | Dako M0755 |
| FoxP3 | Cell Signaling D6O8R |
| CD45RO | ThermoFisher MA1-19452 |
| PanCK A1/3 | Dako M3515 |
| HSA | Merck 264M-9 |
| CD68 | Dako M0876 |
| CD163 | Novocastra NCL-L-CD163 |
| Calprotectin | Dako M0747 |
| MARCO | Sigma HPA063793 |
| FAP | Abcam ab207178 |
| CD90 | Abcam ab92574 |
| αSMA | Dako M0851 clon 1A4 |
| NGFR | Atlas Antibodies HPA004765 |
| COL1A1 | Boster PA214-2 |
| **Secondary Antibodies** | |
| ImPress R | Vector MP-7401 |
| ImPress M | Vector MP-7422-15 |
| Envision M | Dako K4001 |
| Opal HRP Polymer | From Opal 6-Plex Manual Detection Kit |

Multiplex staining sequence for each panel.


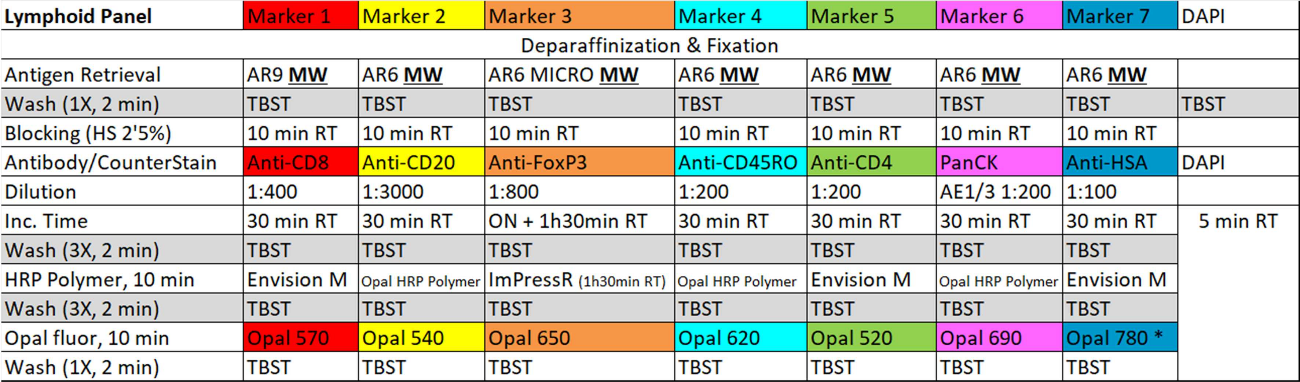

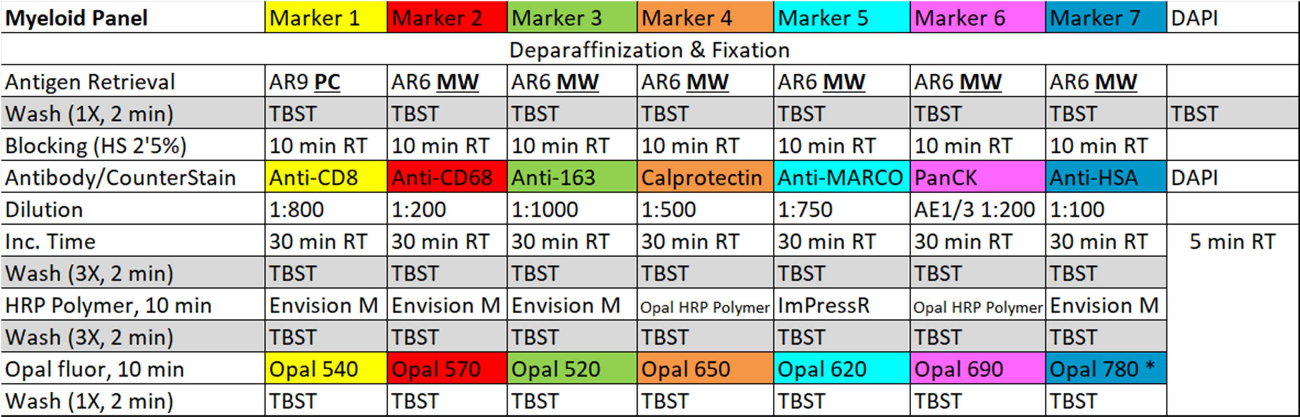

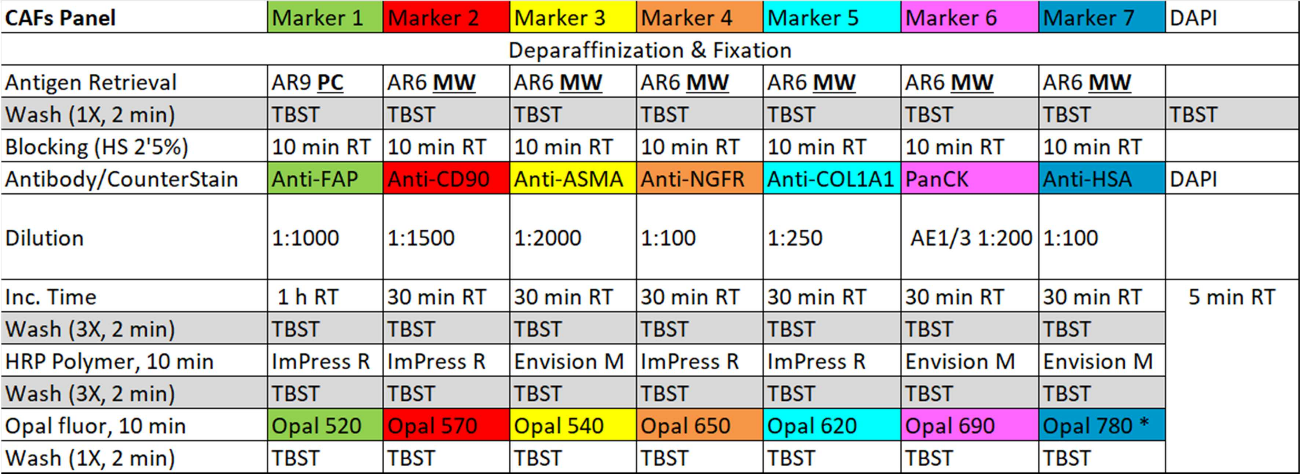


Conventional immunofluorescence

Double/Triple IF allowed us to visualize the expression of two/three different proteins in the same tissue sample by a fluorescence method using Opal dyes (Opal 520, Opal 570 and Opal 650). The staining procedure involved iterative cycles, applying in each cycle the primary antibody (incubation time 30 min, RT) and the secondary polymerised reporter enzyme staining system (undiluted, incubation time 10 min, RT), and followed by tyramide signal amplification and developing with Opal™ fluorophore (1:100, incubation time 10 min, RT) (Akoya Biosciences). After completing each target, HIAR (microwave, pH6, 15 minutes) was used to quench endogenous peroxidase activity, facilitate antigen retrieval, and remove antibodies and polymer system from previous cycle. After final staining cycle 4′,6-diamidino-2-phenylindole (DAPI) was applied to nuclear visualization. Slides were stored at -20 °C until visualization with Zeiss Axio Observer Z1+ Apotome inverted fluorescent microscope and Nikon Eclipse 80i microscope. Images were analyzed using Image J software.

Conventional immunohistochemistry

| **Protein** | **Reference** | **Host** | **Retrieval Buffer** | **Dilution** |
| --- | --- | --- | --- | --- |
| CD90 | Proteintech 66766 | M | EDTA pH9 | 1:40000 |
| CD90 | Abcam ab92574 | R | EDTA pH9 | 1:1000 |
| Calprotectin | Dako M0747 | M | EDTA pH9 | 1:1500 |
| CCR4 | Atlas Antibodies HPA0311613 | R | Sodium Citrate pH6 | 1:40 |
| CD163 | Novocastra NCL-L-CD163 | R | EDTA pH9 | 1:400 |
| CD5L | Abnova MAB22048 | R | EDTA pH9 | 1:200 |
| CD8 | Dako MA5-13473 | M | EDTA pH9 | 1:400 |
| FAPα | Abcam ab227703 | R | EDTA pH9 | 1:100 |
| Collagen 1 | Cell Signaling CS66948 | M | EDTA pH9 | 1:2000 |
| Collagen 1 | BOSTER PA2140-2 | R | EDTA pH9 | 1:100 |
| MARCO | Sigma HPA063793 | R | EDTA pH9 | 1:500 |
| Myeloperoxidase | Cell Signaling CS14569 | R | Sodium Citrate pH6 | 1:1000 |
| Periostin | Abcam ab79946 | R | Sodium Citrate pH6 | 1:50 |
| pSMAD2 | Cell Signalling CS3101 | R | EDTA pH9 | 1:300 |
| αSMA | Dako 1A4 M0851 | M | Sodium Citrate pH6 | 1:400 |
| CD15 | Ventana 760-2504 | M | Sodium Citrate pH6 | prediluted |

Myeloperoxidase, CCR4 was assessed on the TMA samples. Positive cells were counted manually in each of the TMA cores taking into account the total tumoral tissue (both tumor and stroma together) excluding normal adjacent liver.

Staining for pSMAD2 was homogeneous in each tissue core. pSMAD2 staining on the TMA samples was scored as nuclear staining on tumor cells, alkaline phosphatase intensity: 0, no staining; 1, mild intensity; 2, moderate intensity; 3 intense intensity. The resulting score was multiplied by ×2 in case of also observing nuclear staining of CAFs. Finally, a mean of the two cores per patient was recorded.

The whole slide cohort was assessed using a semiquantitative score taking into account cell density measuring the tumoral area with Fiji as follows:

CD8: 0, 0 cells per mm^2^, no staining; 1, 1-to-50 cells/mm^2^; 2, 50-to-100 cells/mm^2^; 3, 101-to-300 cells/mm^2^ and 4, >300 cells/mm^2^. The resulting score was multiplied by ×2 in case of also observing >25% of CD8 over the tumor nests.

Myeloperoxidase: 0, 0 cells per mm^2^, no staining; 1, 1-to-25 cells/mm^2^; 2, 26-to-75 cells/mm^2^; 3,

76-to-150 cells/mm^2^ and 4, >150 cells/mm^2^.

Calprotectin: 0, 0 cells per mm^2^, no staining; 1, 1-to-10 cells/mm^2^; 2, 11-to-50 cells/mm^2^; 3, 50-

to-100 cells/mm^2^ and 4, >100 cells/mm^2^.

Double immunohistochemistry has been done following manufacturer instructions, ImmPRESS® Duet Double Staining Polymer Kit Peroxidase/Alkaline Phosphatase (Vector Laboratories MP-7724-15).


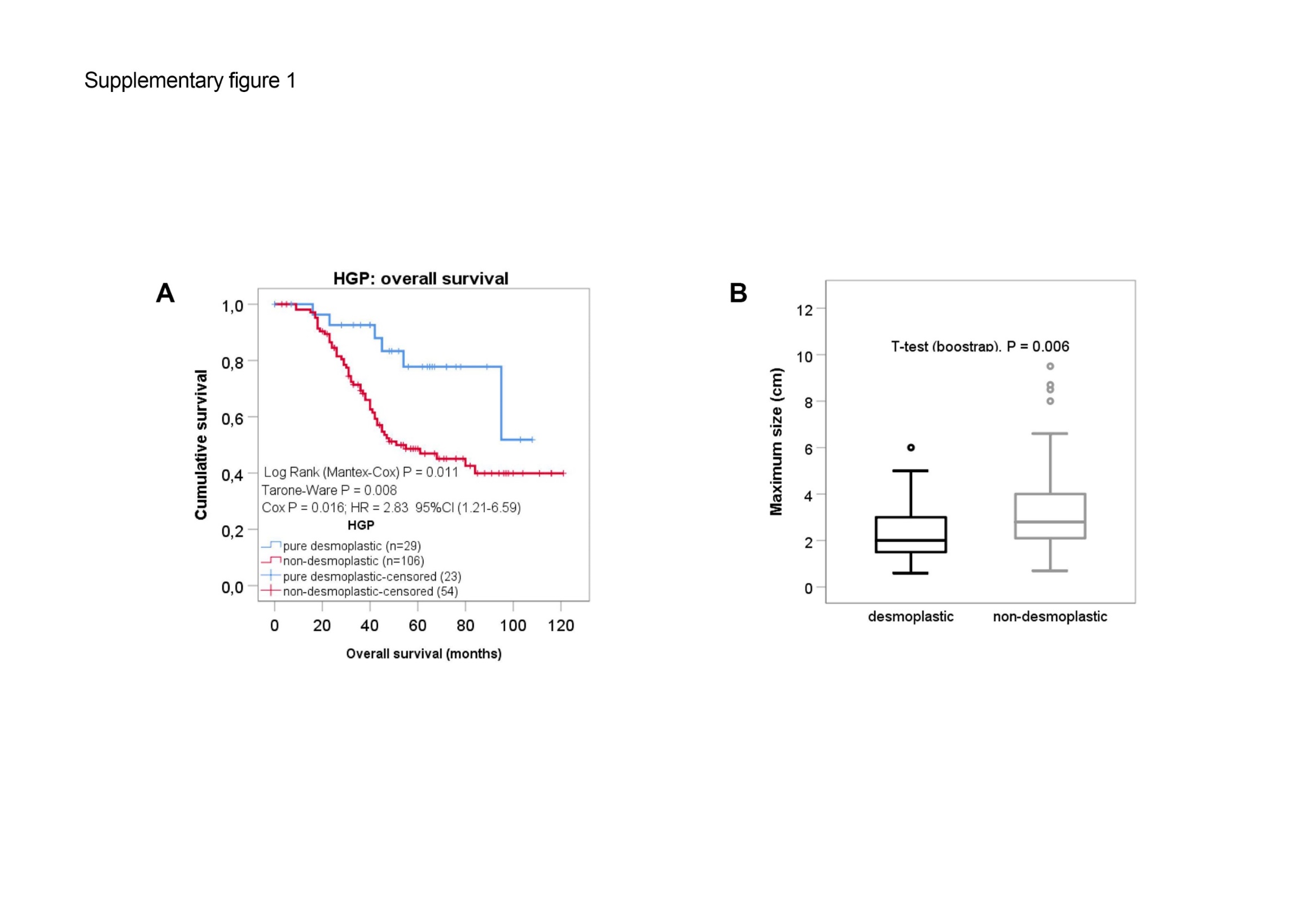


Figure S1: Survival analysis (A), Metastases size (B).

A: Survival analysis (Kaplan-Meier curves and Cox regression) for the 135 patients cohort. Twenty nine patients presented a pure desmoplastic histologic growth pattern, while one hundred six patients have any non-desmoplastic percentage on the tumor-host interface.

B: Desmoplastic/encapsulated metastases displayed a smaller size compared to non-desmoplastic metastases (boostraped T-test, P = 0.006)


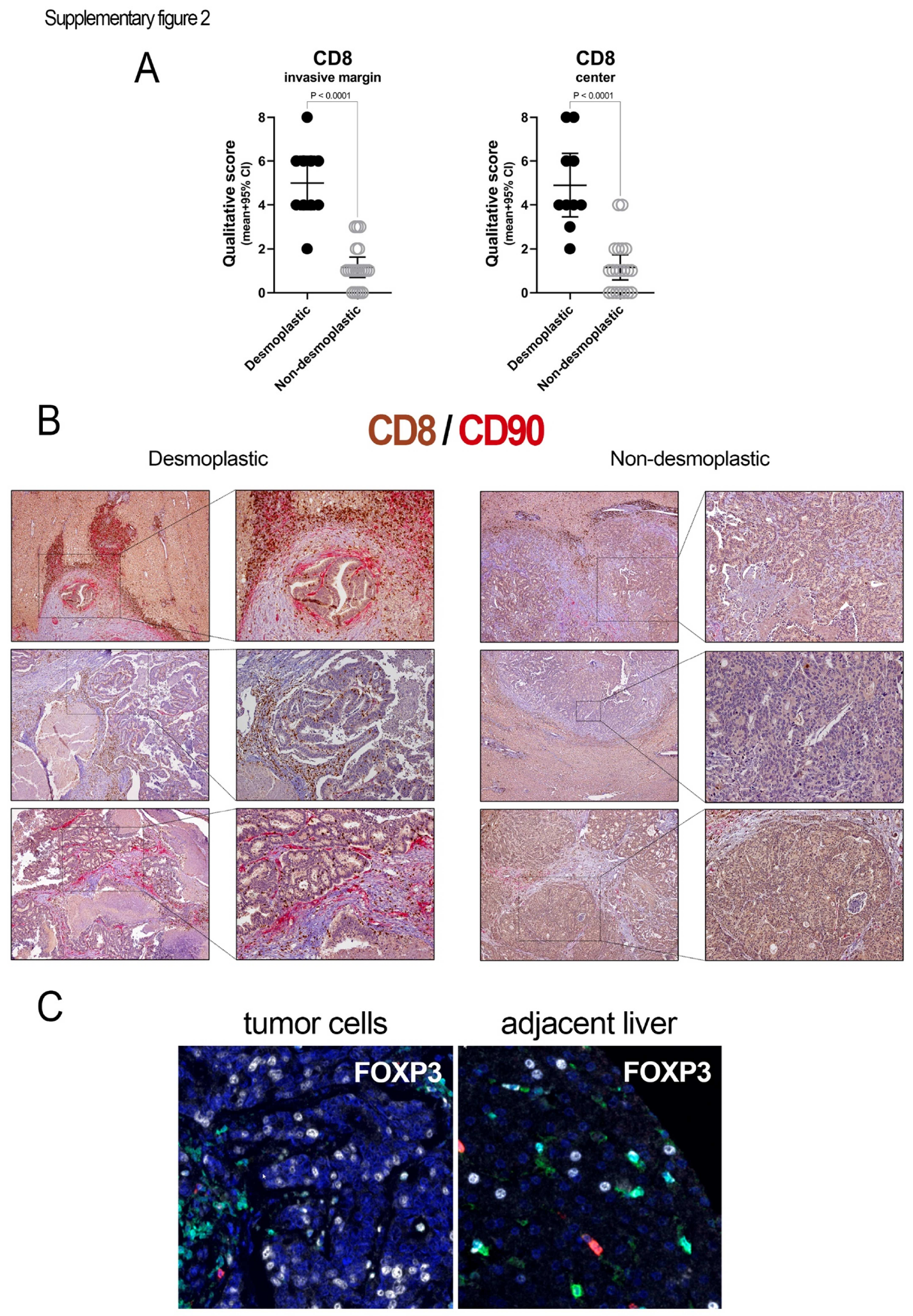


Figure S2

A: Dot-Plot graphs (mean values and 95%CI) of cell densities (cells/mm^2^; black circles for encapsulating metastases, grey circles non-desmoplastic metastases) for the CD8 cells measured in the invasive margin (left) and central areas (right) of the whole slide cohort. Compartments were determined using morphology and segmentation markers (PanCK, Hepatic Specific Antigen and nuclei staining). To compare differences, we used U Mann-Whitney test.

B: Representative images of CD8 (brown) and CD90 (red) staining for an desmoplastic/encapsulating metastasis (dHGP; left image panel) and non-desmoplastic metastasis (non-dHGP, right image panel). As shown in the left image, CD8 cells were more abundant on the dHGP metastasis, both in stromal regions, basically in the fibrous capsule but also inside the tumoral central areas, allocated over the tumor cells (middle and bottom images). On the contrary, on the right image (non-dHGP metastasis), CD8 were mainly retained in the liver and in the tumor liver interface (TLI). Almost no staining was noticed on intratumoral areas neither stromal or tumoral.

C: Nuclear FoxP3 staining (white) on tumor cells (left image) and on hepatocytes from the adjacent normal liver parenchyma (right image).


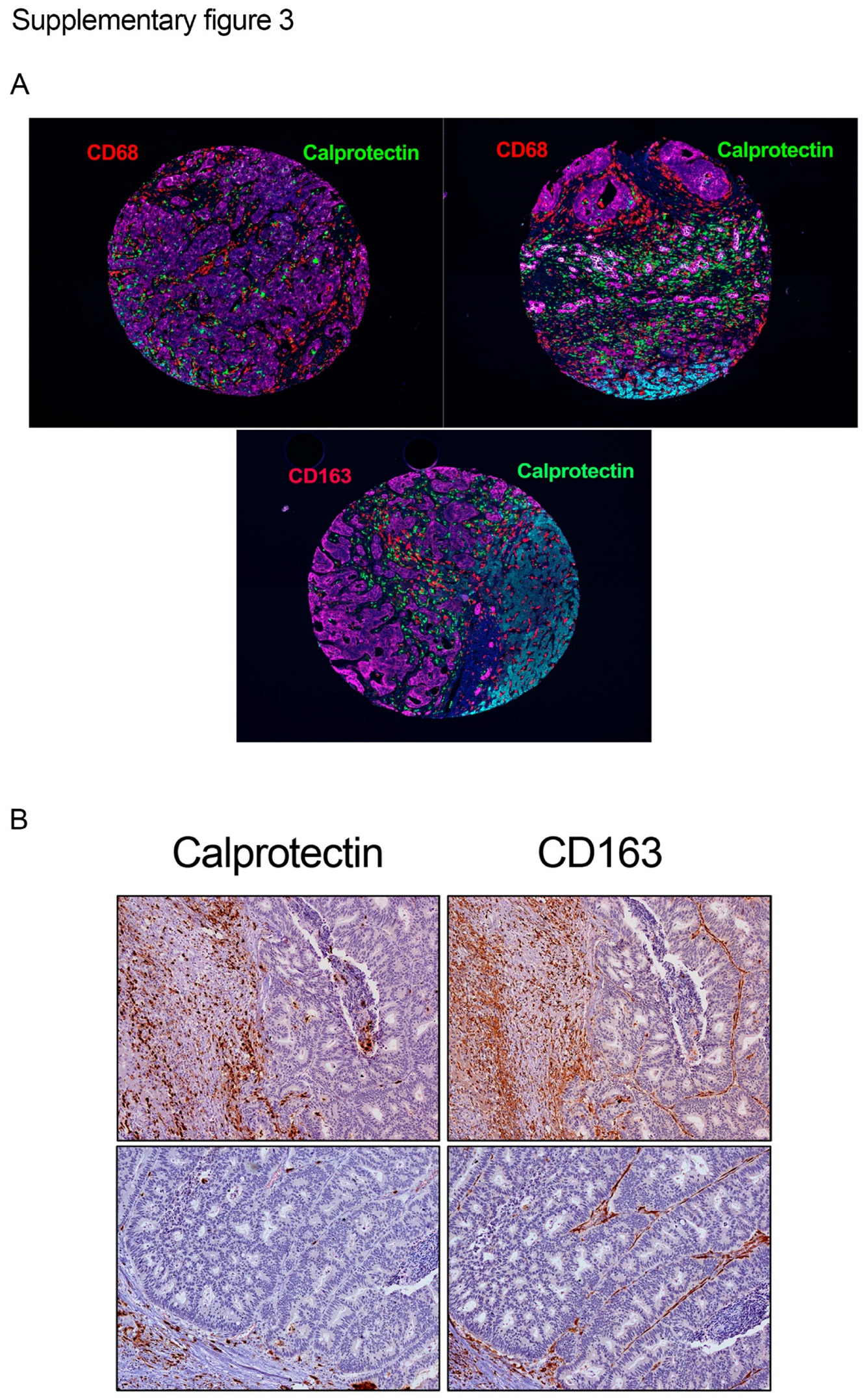


Figure S3: Calprotectin and CD163 staining.

A: Representative multiplex images of different TMA cores showing the phagocytic markers CD68 and CD163 do not overlap with Calprotectin positive cells. Although Calprotectin might be expressed by different cells of the myeloid lineage, as macrophages, this fact seems to not occur in liver metastases from colorectal cancer.

B: Same results were obtained with conventional immunohistochemistry using serial sections in a different whole slide cohort. Calprotectin and CD163 depicted minimal overlap.


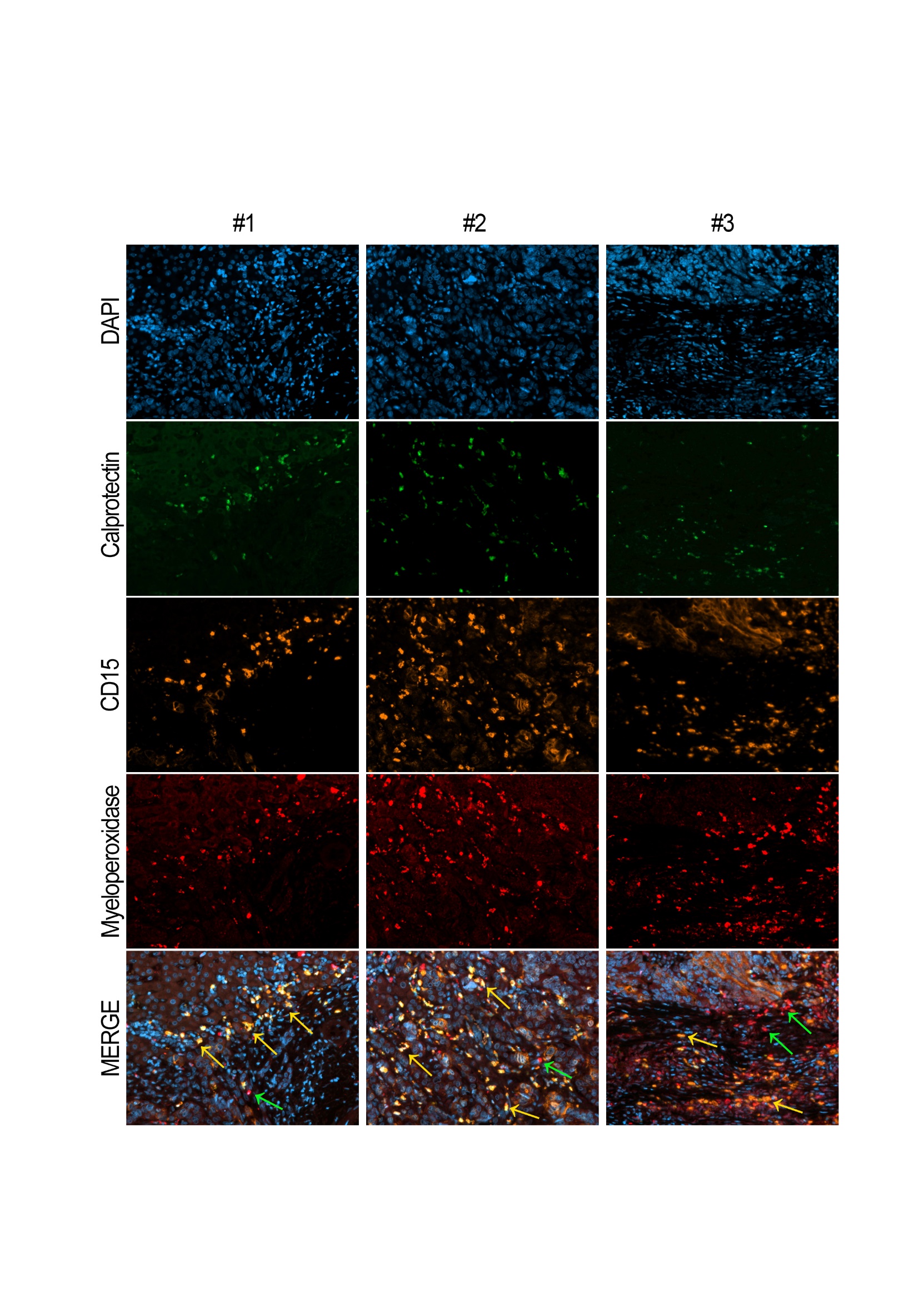
Figure S4: Immunofluorescence staining of myeloid markers.

Immunofluorescence staining of three different liver metastases (#1 and #2 non-dHGP, #3 dHGP, capsule) showing that Calprotectin (nuclear staining), CD15 (membrane staining) and myeloperoxidase (cytoplasmatic staining) are co-expressed in most of the myeloid cells (yellow arrows indicate some examples), probably neutrophils, although given the promiscuity of myeloid markers we can not exclude other cell types as PMN-MDSC. Green arrows indicate myeloperoxidase single staining.

CD15 (fucosyltransferase 4, Lewis X antigen) is a myeloid cell differentiation marker that mediates the adhesion of neutrophils to dendritic cells. CD15 can also be expressed by tumour cells, as displayed in #3.


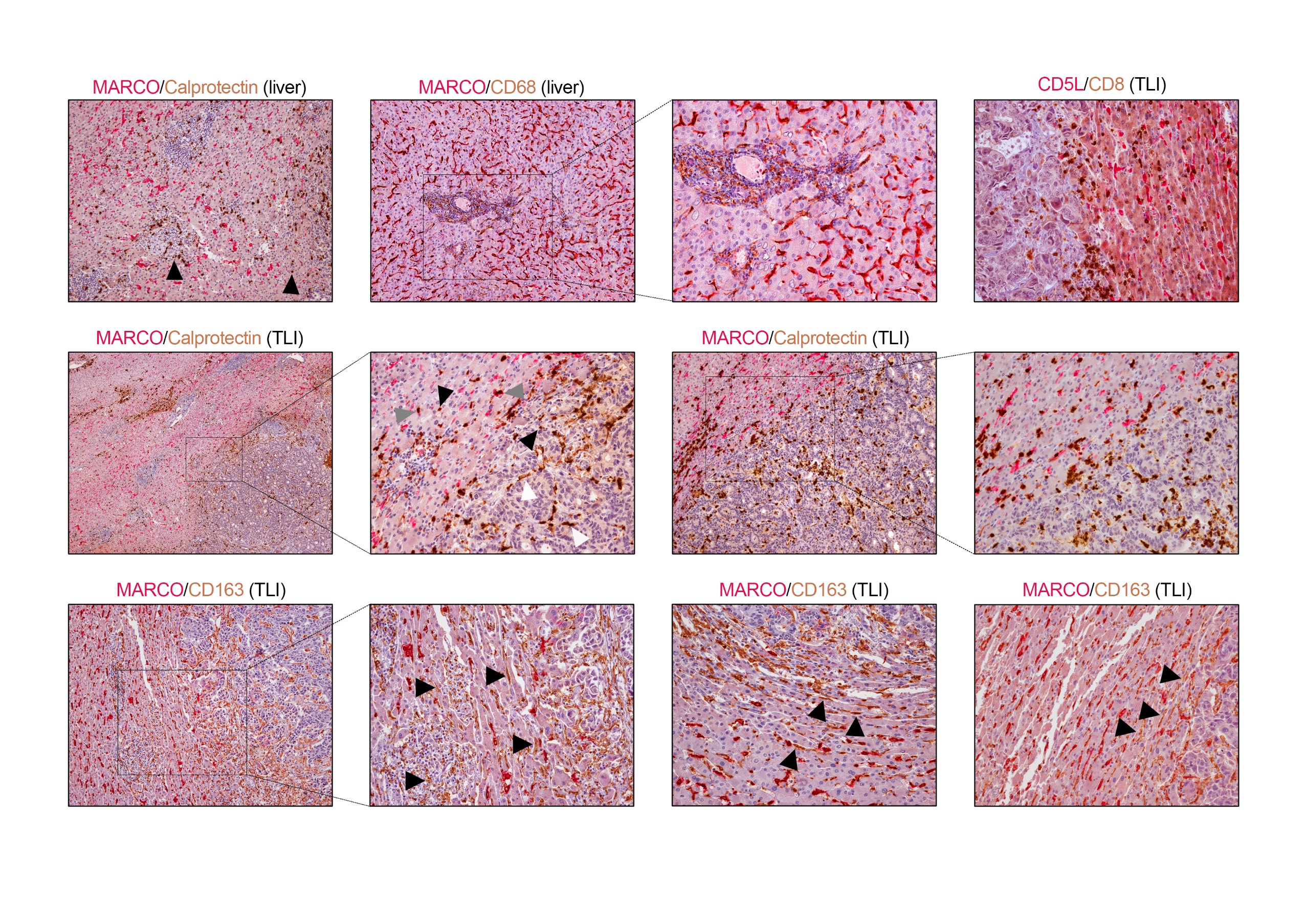
Figure S5: Macrophages and Kupffer cell stainings.

Upper images (from left to right): representative image of a normal adjacent liver showing that MARCO positive cells (red staining) correspond to Kupffer cells, while Calprotectin staining (brown; black arrows, first row) occurs in cells with a rather round morphology and no co-expression with MARCO. The following two images correspond to MARCO (red) and CD68 (brown) staining, showing co-expression on sinusoidal Kupffer cells but illustrated in the magnification image that a subset of portal macrophages do not express MARCO. On the right (first row), CD5L staining (red) showing that this Kupffer cell marker appears not to be found within the tumor. CD5L-positive cells are only seen in the adjacent liver parenchyma. The same is true for MARCO staining (second row). In all the four images of the second row, MARCO staining is only observed in the normal liver parenchyma. As we approach the tumour, some MARCO^+^ cells, that is, Kupffer cells, co-express Calprotectin (grey arrows). However, inside the tumour we observed cells with the same spiculated morphology that express Calprotectin but are MARCO^-^ (white arrows). These cells coexist with other Calprotectin^+^ cells that have a rounded morphology, as are also observed in peritumoral areas of the adjacent parenchyma (black arrows, second row). In the third row, a series of images illustrating the co-expression of MARCO and CD163 in Kupffer cells in the adjacent liver parenchyma. As we get closer to the tumor, in the most proximal areas, the MARCO staining disappears, observing that the cells that infiltrate the tumor only express CD163.


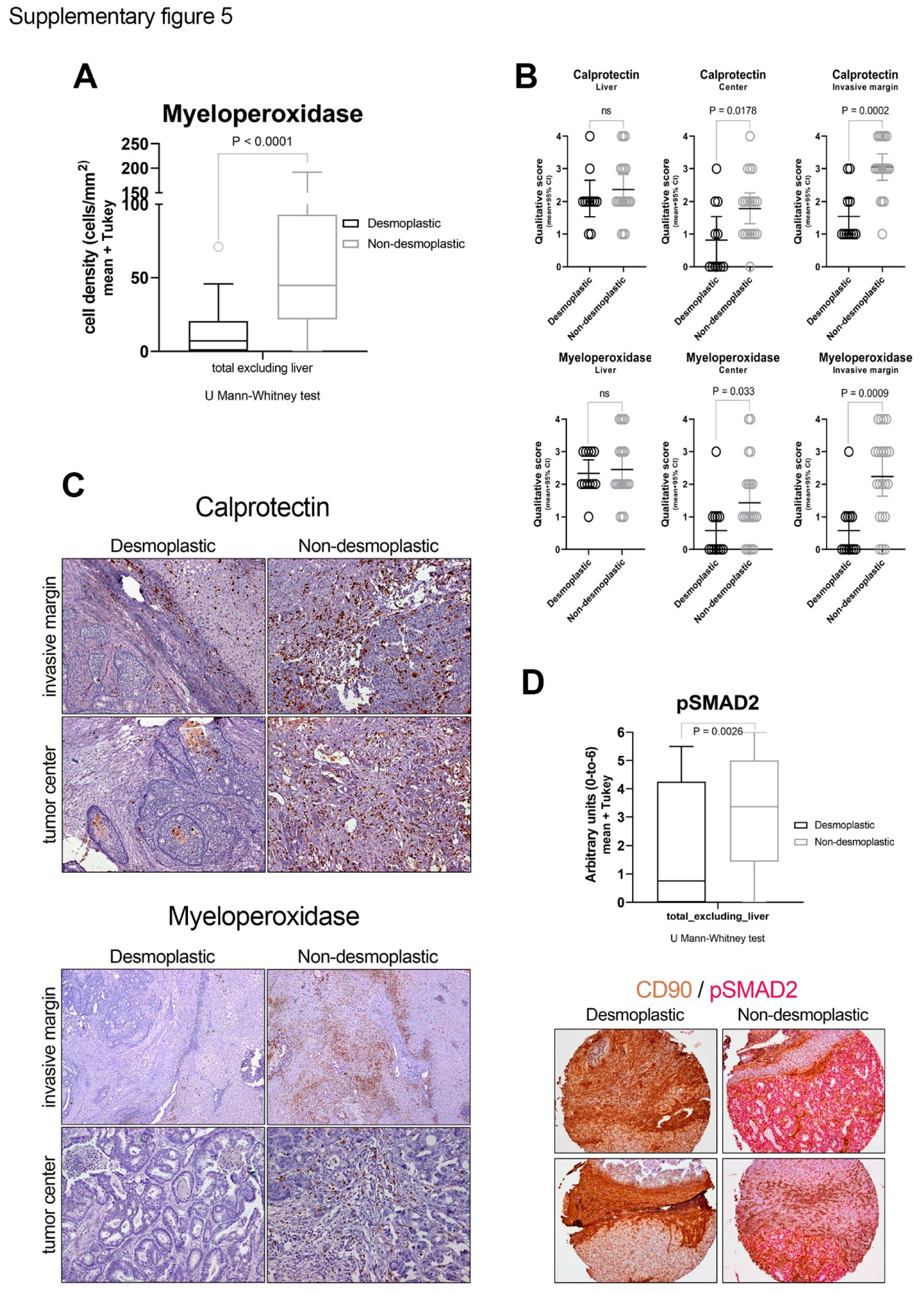


Figure S6

A: Box-plot graphs (mean values and interquartile range) of cell densities (cells/mm^2^; black boxes for encapsulating metastases, grey boxes non-desmoplastic metastases) for Myeloperoxidase and measured in the whole core area considering tumor cells and stromal areas, but excluding adjacent liver. To compare differences, we used U Mann-Whitney with a Pratt correction for zeros and ties.

B: Dot-Plot graphs (mean values and 95%CI) of semiquantitative score (detailed in supplementary methods; cells/mm^2^; black circles for encapsulating metastases, grey circles non-desmoplastic metastases) for Calprotectin and Myeloperoxidase and measured in different compartments (liver, invasive margin, excluding capsule in desmoplastic metastases, and central areas) in the 30 cases of whole slide sections cohort. To compare differences, we used U Mann-Whitney test.

C: Representative images of Calprotectin and Myeloperoxidase staining in central areas and invasive margin of desmoplastic and non-desmoplastic metastases.

D: top panel, box-plot graphs (mean values and interquartile range) of semiquantitative score (detailed in supplementary methods; black boxes for encapsulating metastases, grey boxes non-desmoplastic metastases) for pSMAD2 and measured in the whole core area considering tumor cells and stromal areas, but excluding adjacent liver. To compare differences, we used U Mann-Whitney with a Pratt correction for zeros and ties. Lower panel, representative images of different TMA cores, showing pSMAD2 staining (red) as well as CD90 (brown; carcinoma-associated fibroblasts and portal fibroblasts).

## Figure S7: multiplex staining of CAFs markers αSMA and FAP.


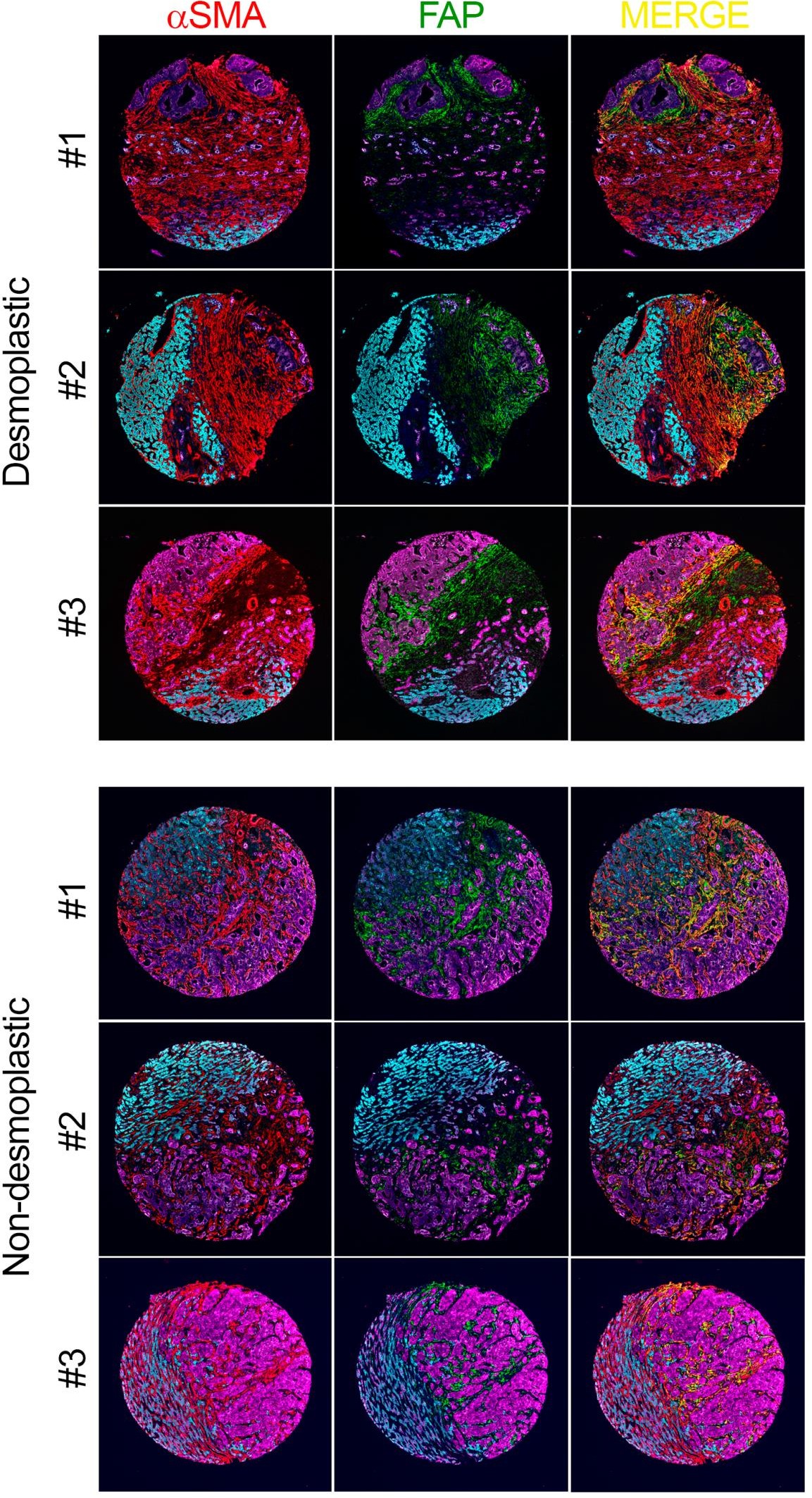


Multispectral images of three desmoplastic liver metastases (upper nine images) and three non- desmoplastic liver metastases (bottom nine images) stained for αSMA (red), FAP (green). In dHGP metastases the capsule is completely stained with αSMA while FAP seems to be arranged as an expression gradient, being mostly expressed on the inner part of the capsule or being exclusively expressed in intratumoural stromal locations. The adjacent liver is stained in cyan (HSA, hepatic specific antigen) and tumour cells in pink (Pancytokeratin).


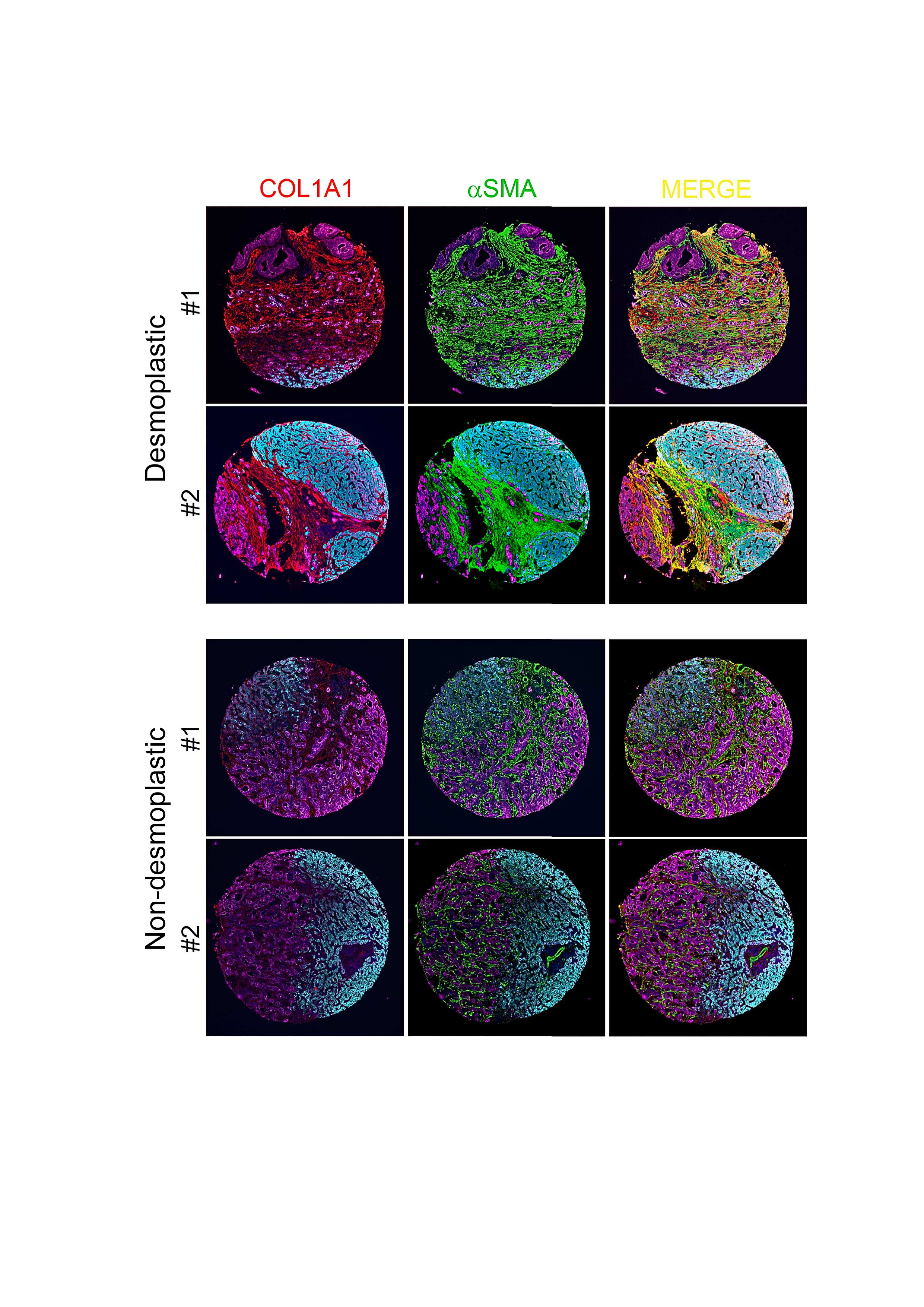
Figure S8: multiplex staining of CAFs markers αSMA and Collagen 1A1

Multispectral images of two desmoplastic liver metastases (upper six images) and two non- desmoplastic liver metastases (bottom six images) stained for Collagen 1 (red) and αSMA (green). In dHGP metastases the capsule and stromal regions around tumour niches are co- stained with Collagen I (COL1A1; red) antibody and αSMA (green) while Collagen I staining is much more discrete in non-encapsulated metastases. The adjacent liver is stained in cyan (HSA, hepatic specific antigen) and tumour cells in pink (Pancytokeratin).

# Figure S9

SupplementarySupplementaryFigurefigure8 9


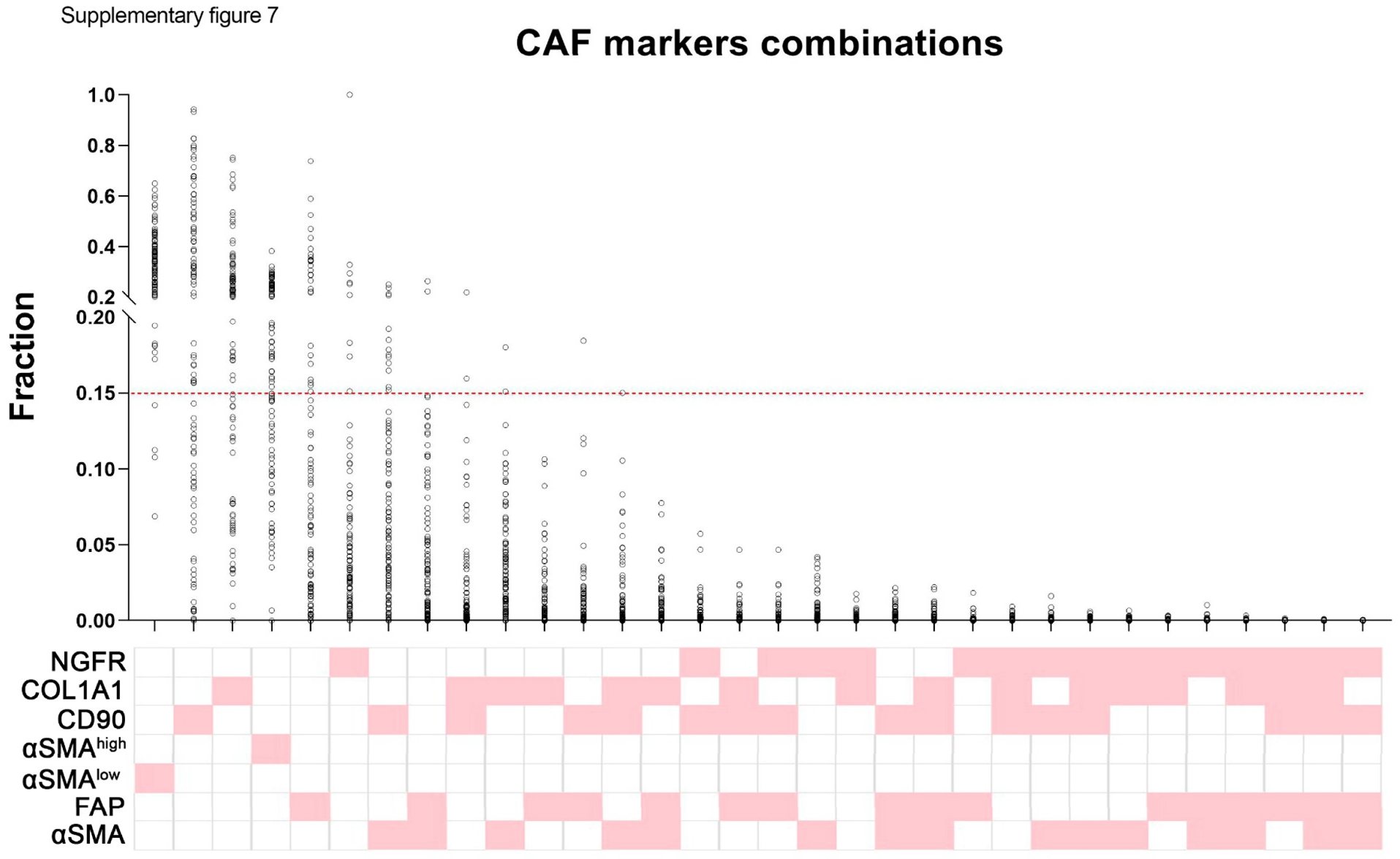


Scatter plot representation of relative density (fractions) of different combinations of CAF markers used. We considered for further analysis those having at lest a fraction > 0.15 in any of the TMA samples.

## Figure S10: immunofluorescence staining of CAFs markers.

Supplementary Figure 9


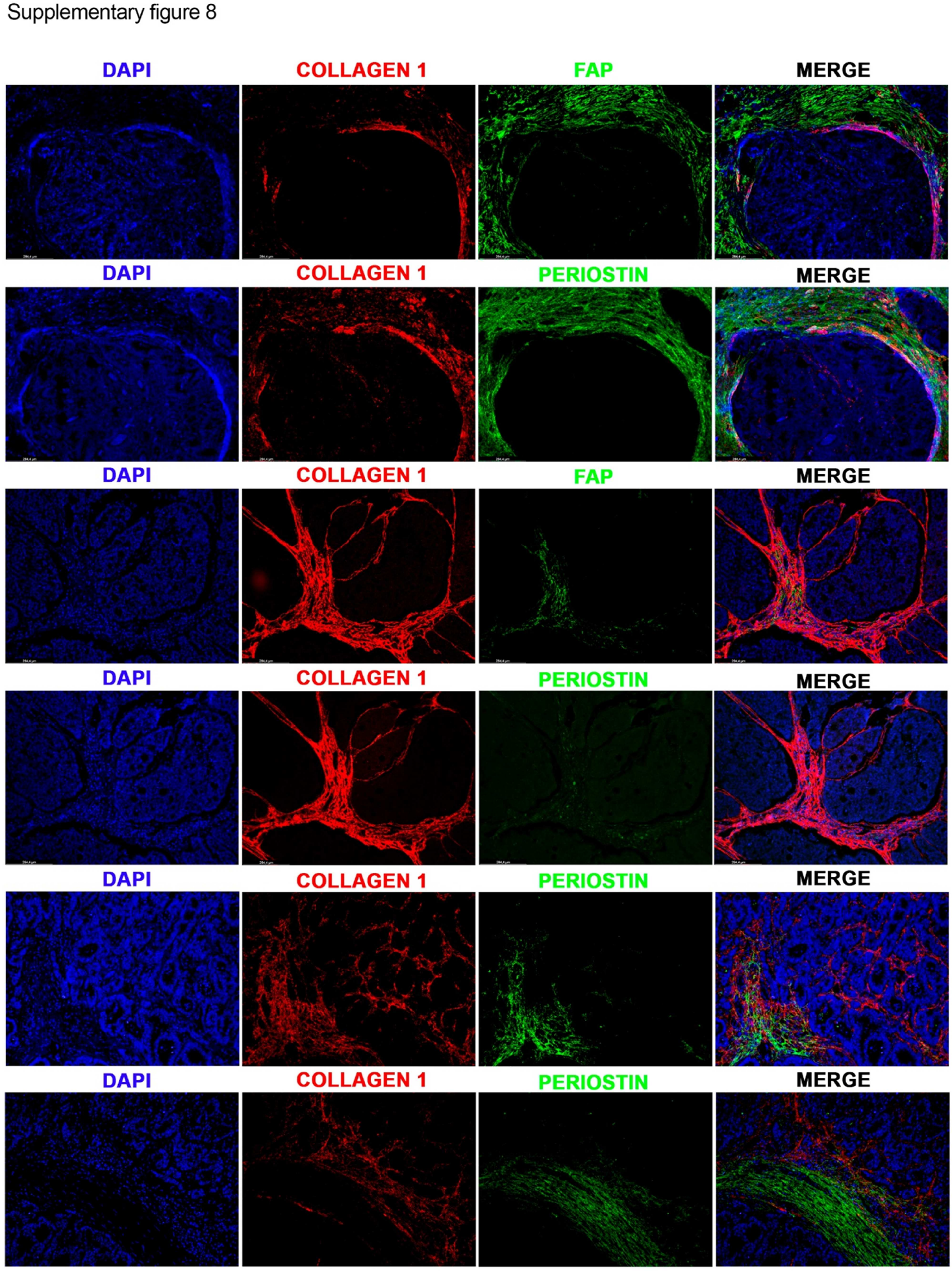


Representative immunofluorescence stainings for Collagen, FAP and Periostin using Opal dyes. The image sequence is intended to demonstrate the coexistence of at least two subpopulations of myCAFs. The first subpopulation, which has been associated with restraining CAFs in different publications, would be ECM-CAFs characterized by the expression of Collagen 1 but not of FAP or Periostin. These last two markers (FAP and Periostin) would be defining a subpopulation of ECM-CAFs that some authors have associated with protumoral functionalities. As shown in the images, the overlap of these two subsets is minimal.


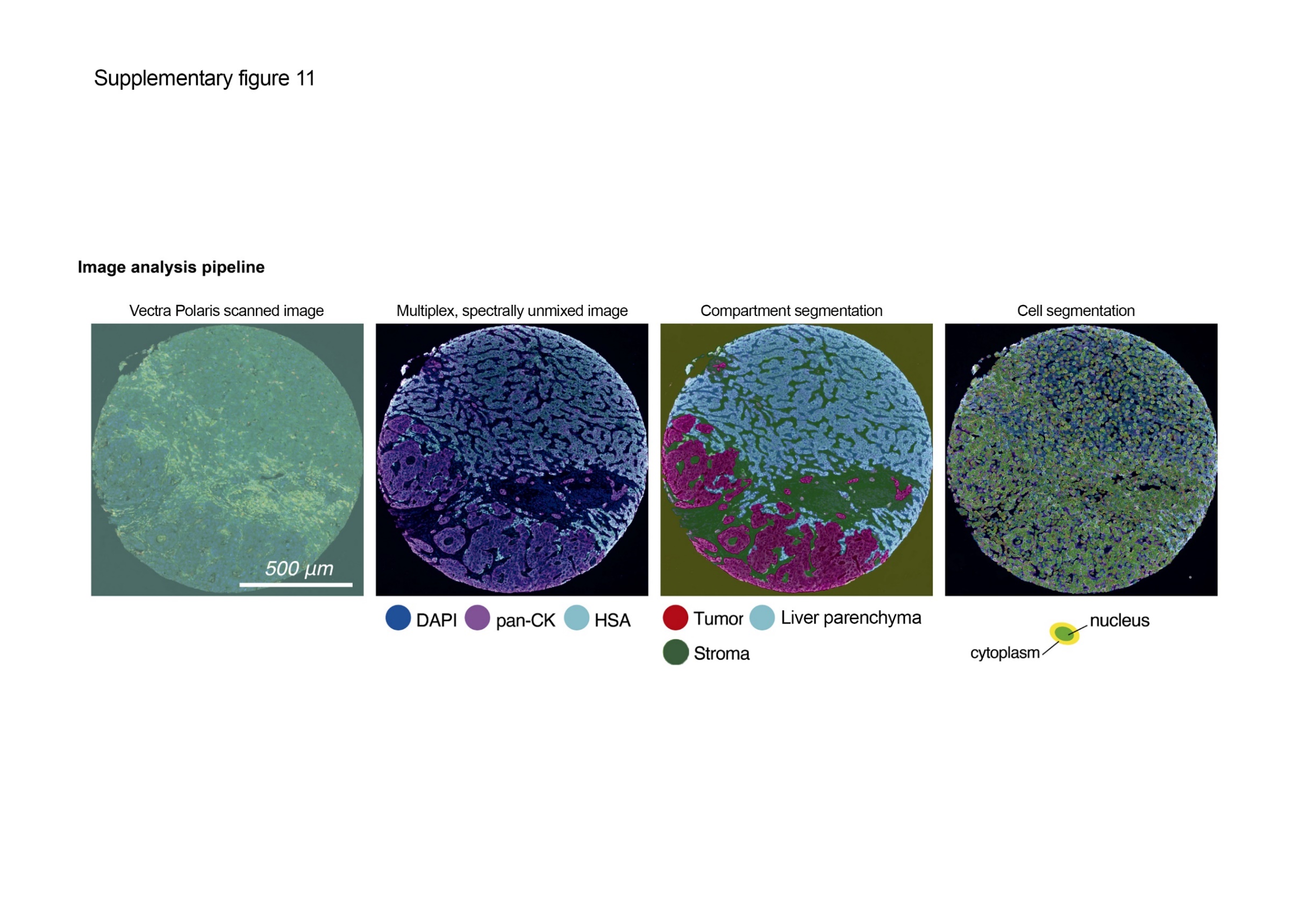
Figure S11: Image analysis pipeline.

Image analysis pipeline: after scanning each TMA core, we performed a compartment segmentation using segmentation markers PanCK (purple) and Hepatic Specific Antigen (HSA; cyan). DAPI was used for cell segmentation. The area at 3 µm (6 pixels) around the nuclear border was considered the cytoplasm area. Finally, all images were reviewed manually to exclude artifacts, necrotic regions, staining defects, bile ducts, portal tracks, damaged tissue or wrongly segmented areas.

Table S1. Association of the clinical variables with the HGP in the 135 patients cohort.

|  | desmoplastic (dHGP) | non desmoplastic (ndHGP) | Chi Square p value |
| --- | --- | --- | --- |
| Gender |  |  |  |
| man | 23 (18.8%)  79.3 | 80 (81.3%)  75.5% | 0.667 |
| woman | 6 (20.7%)  20.7% | 26 (24.5%)  24.5% |  |
| Status |  |  |  |
| alive | 19 (29.7%)  65.5% | 45 (70.3%)  42.5% | 0.022 |
| dead with cancer | 6 (10.2%)  20.7% | 53 (89.8%)  50% |  |
| dead no cancer | 4 (40%)  13.8% | 6 (60%)  5.7% |  |
| lost | 0  0% | 2  1.9% |  |
| Adjuvant treatment metastases |  |  |  |
| yes | 12 (16.2%)  48% | 62 (83.8%)  68.1% | 0.064 |
| no | 13 (31%)  52% | 29 (69%)  31.9% |  |
| Type of metastases |  |  |  |
| synchronous | 15 (36.6%)  51.7% | 26 (63.4%)  24.5% | 0.005 |
| metachronous | 14 (14.9%)  48.3% | 80 (85.1%)  75.5% |  |
| Size (3 cm) |  |  |  |
| < 3 cm | 20 (26.7%)  69% | 55 (73.3%)  51.9% | 0.101 |
| > 3 cm | 9 (15%)  31% | 51 (85%)  48.1% |  |
| Location primary tumour |  |  |  |
| colon | 20 (23%)  74.1% | 67 (77%)  63.8% | 0.316 |
| rectum | 7 (15.6%)  25.9% | 38 (84.8%)  36.2% |  |
| Invasive margin |  |  |  |
| yes | 4 (16.7%)  13.8% | 20 (83.3%)  19.2% | 0.501 |
| no | 25 (22.9%)  86.2% | 84 (77.1%)  80.8% |  |
| Adjuvant treatment primary |  |  |  |
| yes | 12 (14.5%)  41.4% | 71 (85.5%)  67% | 0.012 |
| no | 17 (32.7%)  58.6% | 35 (67.3%)  33% |  |

# Table S2

Main lymphoid cells

|  |  | **p value** | **Adjustment multiple testing** | |  |
| --- | --- | --- | --- | --- | --- |
| **Cell type** | **Location** | **U Mann-Whitney** | **p.adj**  **(Bonferroni)** | **FDR** | **Higher in** |
| **CD4** | Total tissue | 0.475 | 1 | 0.662 | nd |
| **CD4** | Total excluding liver | 0.647 | 1 | 0.662 | nd |
| **CD4** | Tumor | 0.662 | 1 | 0.662 | nd |
| **CD4** | Stroma | 0.621 | 1 | 0.662 | nd |
| **CD4** | Liver | 0.146 | 0.73 | 0.662 | nd |
| **CD8** | Total tissue | 0.009 | **0.045** | **0.021** | **dHGP** |
| **CD8** | Total excluding liver | 0.010 | **0.045** | **0.021** | **dHGP** |
| **CD8** | Tumor | 0.018 | **0.045** | **0.023** | **dHGP** |
| **CD8** | Stroma | 0.231 | 0.23 | 0.231 | nd |
| **CD8** | Liver | 0.013 | **0.045** | **0.021** | **non-dHGP** |
| **CD20** | Total tissue | 0.761 | 1 | 0.761 | nd |
| **CD20** | Total excluding liver | 0.643 | 1 | 0.761 | nd |
| **CD20** | Tumor | 0.255 | 1 | 0.425 | nd |
| **CD20** | Stroma | 0.255 | 1 | 0.425 | nd |
| **CD20** | Liver | 0.002 | **0.012** | **0.012** | **non-dHGP** |
| **FoxP3** | Total tissue | 0.011 | **0.043** | **0.027** | **dHGP** |
| **FoxP3** | Total excluding liver | 0.008 | **0.039** | **0.027** | **dHGP** |
| **FoxP3** | Tumor | 0.121 | 0.24 | 0.151 | nd |
| **FoxP3** | Stroma | 0.080 | 0.24 | 0.133 | nd |
| **FoxP3** | Liver | 0.494 | 0.49 | 0.494 | nd |
| **CD45RO** | Total tissue | 0.397 | 0.84 | 0.397 | nd |
| **CD45RO** | Total excluding liver | 0.324 | 0.84 | 0.397 | nd |
| **CD45RO** | Tumor | 0.281 | 0.84 | 0.397 | nd |
| **CD45RO** | Stroma | 0.037 | 0.15 | 0.093 | nd |
| **CD45RO** | Liver | 0.000 | **0.001** | **0.001** | **non-dHGP** |

|  |  | **p value** | **Adjustment multiple testing** | |  |
| --- | --- | --- | --- | --- | --- |
| **Cell type** | **Location** | **U Mann-Whitney** | **p.adj**  **(Bonferroni)** | **FDR** | **Higher in** |
| **CD4_Single** | Total tissue | 0.298 | 1 | 0.670 | nd |
| **CD4_Single** | Total excluding liver | 0.402 | 1 | 0.670 | nd |
| **CD4_Single** | Tumor | 0.674 | 1 | 0.843 | nd |
| **CD4_Single** | Stroma | 0.881 | 1 | 0.881 | nd |
| **CD4_Single** | Liver | 0.174 | 0.87 | 0.670 | nd |
| **CD4_Mem** | Total tissue | 0.825 | 1 | 0.825 | nd |
| **CD4_Mem** | Total excluding liver | 0.698 | 1 | 0.825 | nd |
| **CD4_Mem** | Tumor | 0.137 | 0.55 | 0.342 | nd |
| **CD4_Mem** | Stroma | 0.223 | 0.67 | 0.372 | nd |
| **CD4_Mem** | Liver | 0.012 | 0.059 | 0.059 | nd |
| **CD4_Treg** | Total tissue | 0.358 | 1 | 0.530 | nd |
| **CD4_Treg** | Total excluding liver | 0.424 | 1 | 0.530 | nd |
| **CD4_Treg** | Tumor | 0.290 | 1 | 0.530 | nd |
| **CD4_Treg** | Stroma | 0.634 | 1 | 0.634 | nd |
| **CD4_Treg** | Liver | 0.123 | 0.62 | 0.530 | nd |
| **CD8_Single** | Total tissue | 0.006 | **0.032** | **0.017** | **dHGP** |
| **CD8_Single** | Total excluding liver | 0.007 | **0.032** | **0.017** | **dHGP** |
| **CD8_Single** | Tumor | 0.018 | **0.038** | **0.022** | **dHGP** |
| **CD8_Single** | Stroma | 0.164 | 0.16 | 0.164 | nd |
| **CD8_Single** | Liver | 0.013 | **0.038** | **0.021** | **non-dHGP** |
| **CD8_Mem** | Total tissue | 0.495 | 1 | 0.495 | nd |
| **CD8_Mem** | Total excluding liver | 0.422 | 1 | 0.495 | nd |
| **CD8_Mem** | Tumor | 0.354 | 1 | 0.495 | nd |
| **CD8_Mem** | Stroma | 0.244 | 0.98 | 0.495 | nd |
| **CD8_Mem** | Liver | 0.044 | 0.22 | 0.220 | nd |

Lymphoid subsets

| **Cell Type** | **Location** | **p value** | **Adjustment multiple testing** | | **Higher in** |
| --- | --- | --- | --- | --- | --- |
|  |  | **U Mann-Whitney** | **p.adj (Bonferroni)** | **FDR** |  |
| **CD163** | Liver | 0.017 | 0.063 | **0.031** | non−dHGP |
| **CD163** | Stroma | 0.007 | **0.020** | **0.009** | non−dHGP |
| **CD163** | Total excluding liver | 0.043 | 0.086 | 0.057 | nd |
| **CD163** | Total | 0.043 | 0.098 | 0.058 | nd |
| **CD163** | Tumor | 0.089 | 0.270 | 0.124 | nd |
| **CD68** | Liver | 0.023 | 0.063 | **0.031** | non−dHGP |
| **CD68** | Stroma | 0.000 | **0.002** | **0.002** | non−dHGP |
| **CD68** | Total excluding liver | 0.010 | **0.029** | **0.019** | non−dHGP |
| **CD68** | Total | 0.033 | 0.098 | 0.058 | nd |
| **CD68** | Tumor | 0.093 | 0.270 | 0.124 | nd |
| **MARCO** | Liver | 0.916 | 0.920 | 0.916 | nd |
| **MARCO** | Stroma | 0.442 | 0.440 | 0.442 | nd |
| **MARCO** | Total excluding liver | 0.159 | 0.160 | 0.159 | nd |
| **MARCO** | Total | 0.498 | 0.500 | 0.498 | nd |
| **MARCO** | Tumor | 0.217 | 0.270 | 0.217 | nd |
| **Calprotectin** | Liver | 0.016 | 0.063 | **0.031** | non−dHGP |
| **Calprotectin** | Stroma | 0.007 | **0.020** | **0.009** | non−dHGP |
| **Calprotectin** | Total excluding liver | 0.004 | **0.018** | **0.018** | non−dHGP |
| **Calprotectin** | Total | 0.007 | **0.029** | **0.029** | non−dHGP |
| **Calprotectin** | Tumor | 0.002 | **0.009** | **0.009** | non−dHGP |

Main myeloid marker

Main myeloid subsets

| **Cell Type** | **Location** | **p value** | **Adjustment multiple testing** | | **Higher in** |
| --- | --- | --- | --- | --- | --- |
|  |  | **U Mann-Whitney** | **p.adj (Bonferroni)** | **FDR** |  |
| **Calprotectin_single** | Tumor | 0.003 | **0.011** | **0.011** | non−dHGP |
| **Calprotectin_single** | Total | 0.009 | **0.037** | **0.026** | non−dHGP |
| **Calprotectin_single** | Total excluding liver | 0.005 | **0.019** | **0.019** | non−dHGP |
| **Calprotectin_single** | Stroma | 0.007 | **0.022** | **0.010** | non−dHGP |
| **Calprotectin_single** | Liver | 0.019 | 0.057 | **0.028** | nd |
| **M1-macrophages** | Tumor | 0.095 | 0.290 | 0.191 | nd |
| **M1-macrophages** | Total | 0.060 | 0.073 | 0.060 | nd |
| **M1-macrophages** | Total excluding liver | 0.010 | **0.029** | **0.019** | non−dHGP |
| **M1-macrophages** | Stroma | 0.000 | **0.002** | **0.002** | non−dHGP |
| **M1-macrophages** | Liver | 0.106 | 0.110 | 0.106 | nd |
| **M2-macrophages** | Tumor | 0.245 | 0.290 | 0.245 | nd |
| **M2-macrophages** | Total | 0.013 | **0.039** | **0.026** | non−dHGP |
| **M2-macrophages** | Total excluding liver | 0.029 | 0.059 | **0.039** | nd |
| **M2-macrophages** | Stroma | 0.007 | **0.022** | **0.010** | non−dHGP |
| **M2-macrophages** | Liver | 0.010 | **0.038** | **0.028** | non−dHGP |
| **Myeloid_non-**  **macrophage** | Tumor | 0.147 | 0.290 | 0.196 | nd |
| **Myeloid_non-**  **macrophage** | Total | 0.037 | 0.073 | **0.049** | nd |
| **Myeloid_non-**  **macrophage** | Total excluding liver | 0.066 | 0.066 | 0.066 | nd |
| **Myeloid_non-**  **macrophage** | Stroma | 0.019 | **0.022** | **0.019** | non−dHGP |
| **Myeloid_non-**  **macrophage** | Liver | 0.021 | 0.057 | **0.028** | nd |

SIA and ratios

| **SIA and macrophage ratios** | **p value** | **Adjustment multiple testing** | | **Higher in** |
| --- | --- | --- | --- | --- |
|  | **U Mann-Whitney** | **p.adj (Bonferroni)** | **FDR** |  |
| **CD8/M2_Total** | 0.001 | **0.009** | **0.009** | dHGP |
| **CD8/M2_Total_excluding_liver** | 0.010 | 0.077 | **0.032** | dHGP |
| **CD8/M2_Tumor** | 0.236 | 0.910 | 0.295 | nd |
| **CD8/M2_Stroma** | 0.005 | **0.048** | **0.027** | dHGP |
| **CD8/M2_Liver** | 0.634 | 0.910 | 0.634 | nd |
| **M1/M2_Total** | 0.167 | 0.910 | 0.244 | nd |
| **M1/M2_Total_excluding_liver** | 0.171 | 0.910 | 0.244 | nd |
| **M1/M2_Tumor** | 0.310 | 0.910 | 0.344 | nd |
| **M1/M2_Stroma** | 0.109 | 0.770 | 0.244 | nd |
| **M1/M2_Liver** | 0.151 | 0.910 | 0.244 | nd |
| **CD8/Myeloid_non-macrophage_Total** | 0.006 | **0.025** | 0.035 | dHGP |
| **CD8/Myeloid_non- macrophage_Total_excluding_liver** | 0.033 | 0.066 | **0.044** | dHGP |
| **CD8/Myeloid_non-macrophage_Tumor** | 0.125 | 0.125 | 0.125 | nd |
| **CD8/Myeloid_non-macrophage_Stroma** | 0.016 | **0.048** | 0.032 | dHGP |
| **CD8/Calprotectin_Total** | 0.00006 | **0.000024** | 0.00024 | dHGP |
| **CD8/Calprotectin_Total_excluding_liver** | 0.000083 | **0.000025** | 0.00016 | dHGP |
| **CD8/Calprotectin_Tumor** | 0.001 | **0.002** | 0.0013 | dHGP |
| **CD8/Calprotectin_Stroma** | 0.002 | **0.002** | 0.002 | dHGP |

Main CAFs and fibroblastic markers

| **Cell Type** | **Location** | **p value** | **Adjustment multiple testing** | | **Higher in** |
| --- | --- | --- | --- | --- | --- |
|  |  | **U Mann-Whitney** | **p.adj (Bonferroni)** | **FDR** |  |
| **FAP** | Total | 0.195 | 1,000 | 0.270 | nd |
| **aSMA** | Total | 0.884 | 1,000 | 0.921 | nd |
| **CD90** | Total | 0.000 | **< 0.001** | < 0.0005 | dHGP |
| **COL1A1** | Total | 0.000 | **0.002** | < 0.001 | dHGP |
| **NGFR** | Total | 0.0061 | **0.031** | 0.0247 | dHGP |
| **FAP** | Total_excluding_liver | 0.139 | 1,000 | 0.217 | nd |
| **aSMA** | Total_excluding_liver | 0.555 | 1,000 | 0.630 | nd |
| **CD90** | Total_excluding_liver | 0.000 | **< 0.001** | < 0.0005 | dHGP |
| **COL1A1** | Total_excluding_liver | 0.000 | **< 0.0005** | < 0.0005 | dHGP |
| **NGFR** | Total_excluding_liver | 0.0063 | **0.031** | 0.0126 | dHGP |
| **FAP** | Tumor | 0.265 | 1,000 | 0.315 | nd |
| **aSMA** | Tumor | 0.004 | 0.073 | **0.012** | non−dHGP |
| **CD90** | Tumor | 0.001 | **0.018** | 0.004 | dHGP |
| **COL1A1** | Tumor | 0.227 | 1,000 | 0.298 | nd |
| **NGFR** | Tumor | 0.0330 | 0.066 | **0.0330** | dHGP |
| **FAP** | Stroma | 0.724 | 1,000 | 0.787 | nd |
| **aSMA** | Stroma | 0.009 | 0.140 | **0.021** | non−dHGP |
| **CD90** | Stroma | 0.017 | 0.190 | **0.028** | dHGP |
| **COL1A1** | Stroma | 0.003 | **0.050** | 0.009 | dHGP |
| **NGFR** | Stroma | 0.0208 | 0.062 | **0.0277** | dHGP |
| **FAP** | Liver | 0.005 | 0.074 | **0.012** | non−dHGP |
| **aSMA** | Liver | 0.000 | **0.010** | 0.002 | non−dHGP |
| **CD90** | Liver | 0.933 | 1,000 | 0.933 | nd |
| **COL1A1** | Liver | 0.247 | 1,000 | 0.309 | nd |
| **NGFR** | Liver | 0.193 | 0.193 | 0.1544 | nd |

|  |  | **p value** | **Adjustment multiple testing** | |  |
| --- | --- | --- | --- | --- | --- |
| **Cell Type** | **Location** | **U Mann- Whitney** | **p.adj(Bonferroni)** | **FDR** | **Higher in** |
| **aSMA^low^_single** | Total | **0.045** | 1 | 0.078 | nd |
| **aSMA^high^_ single** | Total | **3.948E-04** | **0.017** | **0.001** | non−dHGP |
| **NGFR_single** | Total | **0.048** | 1 | 0.081 | nd |
| **CD90_single** | Total | **8.540E-05** | **0.004** | **0.001** | dHGP |
| **COL1A1_single** | Total | **0.009** | 0.280 | **0.019** | nd |
| **FAP_single** | Total | 0.469 | 1 | 0.577 | nd |
| **aSMA_ CD90** | Total | **0.003** | 0.110 | **0.007** | nd |
| **FAP_aSMA** | Total | 0.684 | 1 | 0.742 | nd |
| **CD90_COL1A1** | Total | **1.605E-06** | **9.300E-05** | **4.041E-05** | dHGP |
| α**SMA_COL1A1** | Total | **0.038** | 1 | 0.071 | nd |
| **FAP_CD90** | Total | **2.399E-04** | **0.011** | **0.001** | dHGP |
| α**SMA_CD90_COL1A1** | Total | **3.837E-05** | **0.002** | **2.960E-04** | dHGP |
| **aSMA^low^_single** | Total excluding liver | 0.099 | 1 | 0.154 | nd |
| **aSMA^high^_ single** | Total excluding liver | **0.002** | 0.059 | **0.004** | nd |
| **NGFR_single** | Total excluding liver | 0.099 | 1 | 0.154 | nd |
| **CD90_single** | Total excluding liver | **5.831E-05** | **0.003** | **3.823E-04** | dHGP |
| **COL1A1_single** | Total excluding liver | **2.213E-04** | **0.010** | **0.001** | dHGP |
| **FAP_single** | Total excluding liver | 0.341 | 1 | 0.447 | nd |
| **aSMA_ CD90** | Total excluding liver | **0.002** | 0.073 | **0.005** | nd |
| **FAP_aSMA** | Total excluding liver | 0.705 | 1 | 0.742 | nd |
| **CD90_COL1A1** | Total excluding liver | **7.567E-07** | **4.500E-05** | **4.041E-05** | dHGP |
| α**SMA_COL1A1** | Total excluding liver | **0.008** | 0.270 | **0.018** | nd |
| **FAP_CD90** | Total excluding liver | **9.443E-05** | **0.005** | **0.001** | dHGP |
| α**SMA_CD90_COL1A1** | Total excluding liver | **7.663E-06** | **4.200E-04** | **9.042E-05** | dHGP |
| **aSMA^low^_single** | Tumor | **3.536E-04** | **0.015** | **0.001** | non−dHGP |
| **aSMA^high^_ single** | Tumor | **1.459E-04** | **0.007** | **0.001** | non−dHGP |
| **NGFR_single** | Tumor | **0.017** | 0.510 | **0.034** | nd |
| **CD90_single** | Tumor | **0.003** | 0.110 | **0.007** | nd |
| **COL1A1_single** | Tumor | 0.682 | 1 | 0.742 | nd |
| **FAP_single** | Tumor | 0.956 | 1 | 0.956 | nd |
| **aSMA_ CD90** | Tumor | 0.180 | 1 | 0.252 | nd |
| **FAP_aSMA** | Tumor | 0.406 | 1 | 0.510 | nd |
| **CD90_COL1A1** | Tumor | **3.382E-04** | **0.015** | **0.001** | dHGP |
| α**SMA_COL1A1** | Tumor | 0.696 | 1 | 0.742 | nd |
| **FAP_CD90** | Tumor | 0.764 | 1 | 0.791 | nd |
| α**SMA_CD90_COL1A1** | Tumor | **0.004** | 0.120 | **0.008** | nd |
| **aSMA^low^_single** | Stroma | **2.624E-06** | **1.500E-04** | **4.041E-05** | non−dHGP |
| **aSMA^high^_ single** | Stroma | **2.739E-06** | **1.500E-04** | **4.041E-05** | non−dHGP |
| **NGFR_single** | Stroma | 0.622 | 1 | 0.733 | nd |
| **CD90_single** | Stroma | **0.018** | 0.540 | **0.036** | nd |

CAFs subsets

| **COL1A1_single** | Stroma | **0.044** | 1 | 0.078 | nd |
| --- | --- | --- | --- | --- | --- |
| **FAP_single** | Stroma | 0.705 | 1 | 0.742 | nd |
| **aSMA_ CD90** | Stroma | 0.231 | 1 | 0.316 | nd |
| **FAP_aSMA** | Stroma | 0.164 | 1 | 0.241 | nd |
| **CD90_COL1A1** | Stroma | **4.013E-05** | **0.002** | **2.960E-04** | dHGP |
| α**SMA_COL1A1** | Stroma | 0.137 | 1 | 0.208 | nd |
| **FAP_CD90** | Stroma | **0.001** | **0.042** | **0.003** | dHGP |
| α**SMA_CD90_COL1A1** | Stroma | **1.106E-04** | **0.005** | **0.001** | dHGP |
| **aSMA^low^_single** | Liver | **3.953E-04** | **0.017** | **0.001** | non−dHGP |
| **aSMA^high^_ single** | Liver | **1.117E-05** | **0.001** | **1.098E-04** | non−dHGP |
| **NGFR_single** | Liver | 0.259 | 1 | 0.347 | nd |
| **CD90_single** | Liver | 0.677 | 1 | 0.742 | nd |
| **COL1A1_single** | Liver | 0.371 | 1 | 0.476 | nd |
| **FAP_single** | Liver | 0.069 | 1 | 0.114 | nd |
| **aSMA_ CD90** | Liver | 0.906 | 1 | 0.922 | nd |
| **FAP_aSMA** | Liver | **0.024** | 0.700 | **0.046** | nd |
| **CD90_COL1A1** | Liver | 0.168 | 1 | 0.241 | nd |
| α**SMA_COL1A1** | Liver | **0.007** | 0.240 | **0.016** | nd |
| **FAP_CD90** | Liver | NA | NA | NA | nd |
| α**SMA_CD90_COL1A1** | Liver | 0.552 | 1 | 0.665 | nd |

Table S3

|  | **DESMOPLASTIC (dHGP)** | **NON DESMOPLASTIC (non-dHGP)** | **CHI SQUARE p value** |
| --- | --- | --- | --- |
| **Gender** | | | |
| **male** | 19 (86,4,3%) | 59 (75,6%) | 0,284 |
| **female** | 3 (13,6%) | 19 (24,4%) |  |
| **Survival status** | | | |
| **alive** | 10 (45,5%) | 35 (44,9%) | 0,345 |
| **dead with cancer** | 9 (40,9%) | 39 (50%) |  |
| **dead no cancer** | 3 (13,6%) | 3 (3,8%) |  |
| **lost** | 0 | 1 (1,3%) |  |
| **Adjuvant treatment metastases** | | | |
| **yes** | 10 (50%) | 44 (68.8%) | 0,127 |
| **no** | 10 (50%) | 20 (31,3%) |  |
| **Type of metastases** | | | |
| **synchronous** | 10 (45,5%) | 17 (21,8%) | **0,027** |
| **metachronous** | 12 (54,5%) | 61 (78,2%) |  |
| **Size (3 cm)** | | | |
| **< 3 cm** | 14 (63,6%) | 36 (46,2%) | 0,148 |
| **> 3 cm** | 8 (36,4%) | 42 (53,8%) |  |
| **Location primary tumor** | | | |
| **colon** | 16 (76,2%) | 46 (59,7%) | 0,166 |
| **rectum** | 5 (23,8%) | 31 (40,3%) |  |
| **Affected resection margin** | | | |
| **yes** | 2 (9,1%) | 17 (22,1%) | 0,173 |
| **no** | 20 (90,9%) | 60 (77,9%) |  |
| **Adjuvant treatment primary** | | | |
| **yes** | 12 (54,5%) | 51 (67,1%) | 0,279 |
| **no** | 10 (45,5%) | 25 (32,9%) |  |
| **MSI/MSS status** | | | |
| **MSI** | 2 (9,1%) | 8 (10,3%) | 0,872 |
| **MSS** | 20 (90,9%) | 70 (89,7%) |  |

Table S4: attached excel file

Table S5

|  | N | percentage |
| --- | --- | --- |
| Gender |  |  |
| man | 103 | 76,30% |
| woman | 32 | 23,70% |
| Type of metastases |  |  |
| synchronous | 41 | 30,40% |
| metachronous | 94 | 69,60% |
| Location of primary tumor |  |  |
| colon | 87 | 64,40% |
| rectum | 45 | 33,30% |
| lost | 3 | 2,20% |
| Adjuvant treatment primary |  |  |
| yes | 83 | 61,50% |
| no | 52 | 38,50% |
| Adjuvant treatment metastases |  |  |
| yes | 74 | 54,80% |
| no | 42 | 31,10% |
| lost | 19 | 14,10% |
|  |  |  |
| Mean age (years) | 67,32 | range 36-86 |
| Mean size (cm) | 3,08 cm | range 0,6-12,4 |

**iCAF signature**

ADAMTS5

ADH1B

C3

C4B

C7

CCL2

CCL7

COLEC12

CXCL1

CXCL12

CXCL8

LIF

FGF7

GAS1

GSN

CXCL3

IL33

IL6

PCOLCE2

PDGFD

PLPP3

PTGS2

PTX3

RSPO3

SLIT3

CYGB

CFD

DPT

PRDM1

IL11

SVEP1

IL1B

CLEC3B

CSF3

HGF

SAA1

CCL21

FBLN1

FBLN5

EFEMP1

**myCAF signature**

ACTA2

ASPN

BGN

CALD1

CCDC80

CCL11

CNN1

COL11A1

COL15A1

COL1A1

COL1A2

COL3A1

COL4A1

COL6A3

COMP

CRLF1

CTHRC1

ELN

FAP

FN1

HTRA3

ID4

IGFBP3

IGFBP7

INHBA

ISLR

LIPG

LRRC15

MFAP4

MMP23B

POSTN

SERPINE2

SFRP4

SULF1

TAGLN

TGFB1

THBS2

THY1

TNC

TPM1
